# Repeated biocide treatments cause changes to the microbiome of a food industry floor drain biofilm model

**DOI:** 10.3389/fmicb.2025.1542193

**Published:** 2025-03-14

**Authors:** Martin Laage Kragh, Nanna Hulbæk Scheel, Pimlapas Leekitcharoenphon, Lisbeth Truelstrup Hansen

**Affiliations:** National Food Institute, Technical University of Denmark, Kongens Lyngby, Denmark

**Keywords:** disinfect, food environment, antibiotic resistance gene, tolerance, benzalkonium chloride, peracetic acid, sodium hypochlorite, metagenomic

## Abstract

There is a concern about the development of microbial tolerance and resistance to biocides due to their repeated use within the food industry. This study aimed to develop a floor drain biofilm model and test whether repeated biocide treatment would result in increased tolerance to biocides. Culturomics and shotgun metagenomic analysis of 14 drains and 214 bacterial isolates from three industrial food production environments revealed microbiomes with great diversity and complexity, but with the dominance of a few highly abundant taxa, including *Pseudomonas*. A representative drain biofilm was created (3 days, 15°C) using 31 whole genome sequenced bacterial isolates from 24 genera. The biofilm model represented 47–58% and 76–81% of the microbial abundance observed in the metagenome and viable microbiota, respectively. The biofilm model was exposed on days 3 and 6 to water or different industrial concentrations of benzalkonium chloride (BC), peracetic acid (PAA), or sodium hypochlorite (SH). Analysis of the viable survivors using MALDI-TOF MS and the regrowing biofilms using 16S rRNA amplicon sequencing showed how the diversity of the biofilm decreased but without any change in biocide tolerance as seen in log reductions (CFU/cm^2^). The use of different biocides did, however, exert significantly different selective pressures on the microbiomes as *Citrobacter*, *Acinetobacter*, *Aeromonas*, and *Pseudomonas* dominated the biofilm after treatments with SH or PAA, while *Serratia* and *Moraxella* dominated after treatments with BC. The dominance of *Serratia marcescens* could be explained by the carriage of a BC efflux pump (*oqxB*) and the highest (20 mg/L BC) minimum inhibitory concentration (MIC) result of the drain isolates. In contrast, despite carrying a BC efflux pump (*qacH*), *Listeria monocytogenes* ST121 did not show increased survival or presence in the biofilm after BC treatments. Only the highest tested concentration of PAA was able to completely eradicate *L. monocytogenes*. The developed biofilm model and the repeated biocide treatments enabled a better understanding of how biocides affect the biofilm microbiome. Future research should involve testing biocide rotation strategies to control biofilm regrowth and inactivation of persistent foodborne pathogens in floor drains.

## 1 Introduction

Food manufacturers depend on the removal and inactivation of microbes through sanitation programs, including cleaning and disinfection schemes, to maintain a food production environment suitable for producing safe food. Recently, however, there has been increased interest in the potential development of microbial tolerance or adaptation to biocides due to repeated application of the same biocide (Bland et al., 2022a, b; He et al., 2022; Rahman et al., 2022). Case studies with biofilms in hospital sinks and food conveyor belts have shown how difficult eradication of biofilms may be even when using multiple biocide treatments with increasing concentrations (Fagerlund et al., 2020; Ledwoch et al., 2020; Anantharajah et al., 2024). In general, drains and sinks constitute difficult-to-clean sites in both clinical settings and food production. Floor drains are continuously fed with a diverse pool of bacteria that have traveled through the food processing environment (FPE; Dzieciol et al., 2016) after being introduced via different raw materials, ingredients, workers, equipment, and vehicles depending on the quality of zoning within the food processing plant (Berrang et al., 2010; NicAogáin and O’Byrne, 2016). Logically, drains also serve as the exit and collection point of cleaning and biocide residues together with dirt, residual organic matters, and non-viable and viable bacteria, some of which may adhere and form biofilms on surfaces within sinks and drains (Wagner and Stessl, 2014; Dzieciol et al., 2016). Therefore, drains may become a reservoir where pathogenic bacteria and bacteria with strong biofilm-producing capabilities may persist to create opportunities for re-introduction into the FPE through splashing caused by wrongful cleaning and trolleys (Berrang and Frank, 2012; Murugesan et al., 2015; Magdovitz et al., 2020).

The foodborne pathogen *Listeria monocytogenes* is a ubiquitous microbe that may cross-contaminate food products due to its persistence in a food production environment for years (Ferreira et al., 2014; Stessl et al., 2020). Routine sampling for *Listeria* spp. has shown drains to be one of the sites with the highest probability of positive samples (Estrada et al., 2020; Magdovitz et al., 2020; Bardsley et al., 2024). In addition, depending on the microbiota of drains the positive ratio for *Listeria* spp. may increase due to cooperative and competitive interactions within biofilms (Fox et al., 2014; Møretrø and Langsrud, 2017; Fagerlund et al., 2021). In recent years, it has been shown that *L. monocytogenes* persistence may be partly aided by the possession of genes conferring increased tolerance to quaternary ammonium compound (QAC)-based biocides and/or gaining tolerance through repeated exposure to low biocide concentrations (Møretrø et al., 2017; Luque-Sastre et al., 2018; Fagerlund et al., 2022; He et al., 2022). However, a search in the publicly available whole genome sequences of 37,897 *L. monocytogenes* isolates showed that less than 30% carry QAC-tolerant genes, indicating that survival in a food production environment is also affected by other factors (Ivanova et al., 2025). Indeed, the formation of biofilm was more disruptive for the effect of benzalkonium chloride, a QAC biocide, on *L. monocytogenes* isolates than the harborage of one of the QAC-tolerant genes (Kragh et al., 2024). Similar to other pathogens such as *E. coli* O157:H7 and *Salmonella enterica*, survival of *L. monocytogenes* following biocide exposure is aided when embedded in multispecies biofilms dominated by genera such as *Acinetobacter, Pseudomonas, and Stenotrophomonas* (Fagerlund et al., 2017; Dass et al., 2020; Maillard and Centeleghe, 2023; Thomassen et al., 2023a; Rolon et al., 2024). As biofilms interfere with the efficacy of biocides, it is important to use realistic biofilm models when studying the effect of biocide treatments on microbiomes in FPEs and the survival of pathogens such as *L. monocytogenes*.

The era of microbiome and metagenome techniques has enabled studies of the complexity, diversity, and resistome of biofilms in food production environments and the potential spread of biocide tolerance genes and antimicrobial resistance genes (AMR) within biofilms (Alvarez-Molina et al., 2023; Flores-Vargas et al., 2023; Nimaichand et al., 2023; Xu et al., 2023). By comparing the core microbiome among different samples, it is furthermore possible to define the most abundant genera across samples, productions, or specific niches; e.g., drains in beef productions were shown to be dominated by *Pseudomonas*, *Psychrobacter*, and *Acinetobacter* (Palanisamy et al., 2023). This approach opens for the development of realistic and representative biofilm models to study the effect of biocide treatments on these niches in the laboratory.

The objectives of the study were to (i) create a representative drain biofilm model based on culturomics and shotgun metagenomic characterization of floor drains from cheese, shrimp, and fish roe food production environments, (ii) use the representative drain biofilm model to test the effect of repeated biocide treatments on the composition of the surviving and regrowing microbiome and specifically on the survival of *L. monocytogenes*, and (iii) characterize the isolates of the model by biocide MIC testing and whole genome sequencing (WGS) to improve the understanding and evaluation of the observed biofilm results.

## 2 Materials and methods

**Figure 1:**
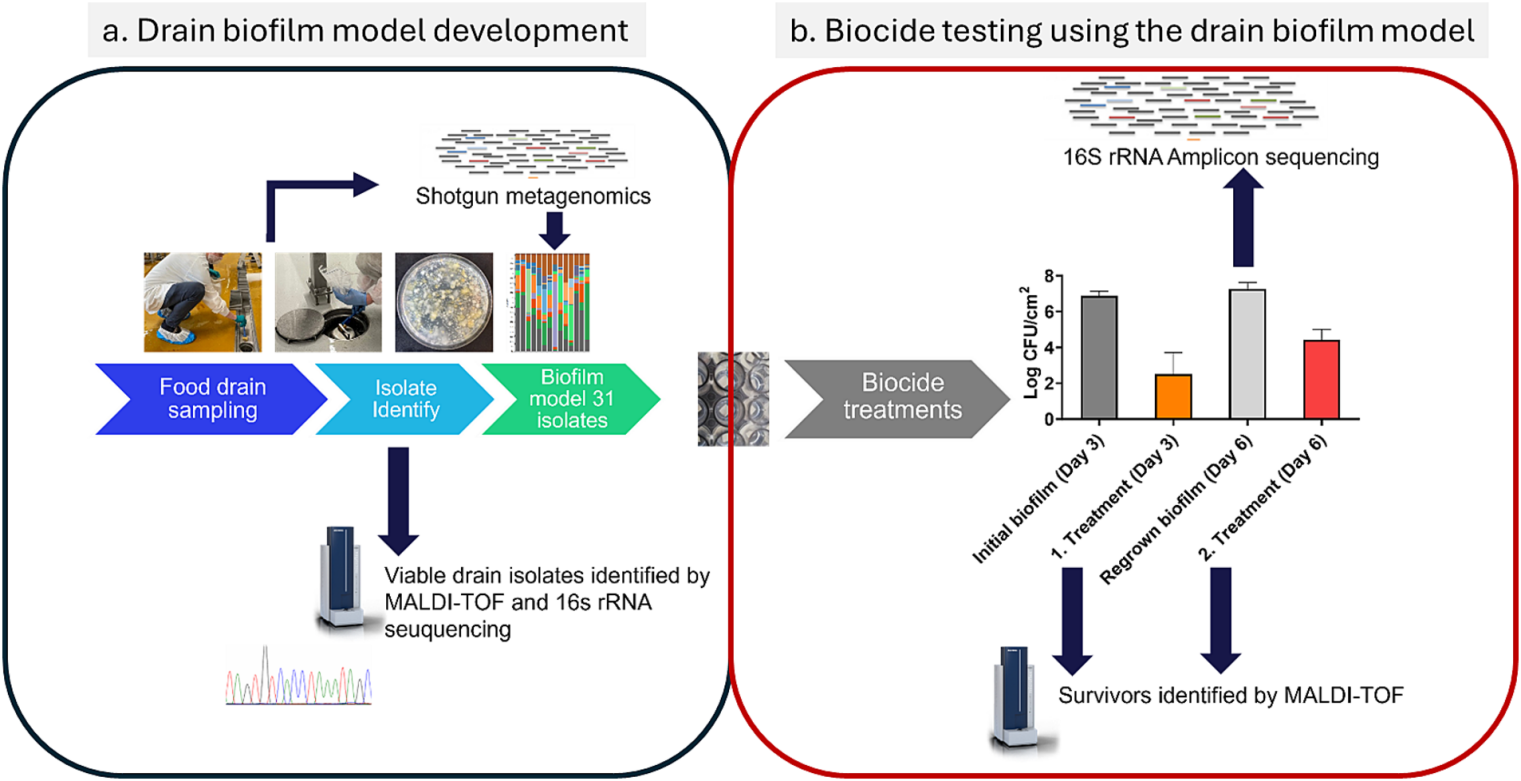
Methodology in brief, showing **(a)** the analysis and selection of representative isolates for the drain biofilm model and **(b)** the scheme for biocide testing using the drain biofilm model and ensuing analysis of the changes in the microbiome caused by the treatments. Refer to Sections 2.1 to Section 2.8 for details.

A brief overview of the methodology can be seen in Figure 1. A representative drain biofilm model was designed based on the characterization of floor drain microbiomes from three different FPEs by metagenomic sequencing and identification of representative isolates from the viable drain microbiota. This resulted in the selection of 31 whole genome sequenced isolates for the drain biofilm model (Figure 1A). For the testing of the effect of biocides, biofilms were formed (3 days, 15°C) on stainless steel coupons before treatments on day 3. Surviving biofilm bacteria were regrown (15°C) for an additional 3 days before being treated on day 6 using the same biocide concentration (Figure 1B). The biofilm microbiomes of the initial biofilm (untreated, day 3) and regrown biofilms (day 6, 3 days after treatment with a biocide) were analyzed using 16S rRNA amplicon sequencing. Survivors were enumerated by plate counts, and colonies were identified using MALDI-TOF before and after the first and second biocide treatments (Figure 1).

### 2.1 Sampling of food production drains

Three food production sites were visited for sampling of drains within food production environments (Table 1). A cheese-producing company was visited two times with 9 months between each visit, whereas a seafood company with separate roe and shrimp production facilities was visited once. Samples from drains (*n* = 14) were taken using industrial surface sampling sponges pre-moistened with Dey/Engley neutralizing buffer (VWR, Søborg, Denmark). From each drain, at least triplicate samples were obtained from individual unique spots of 10 cm^2^. Swaps were transported refrigerated (<5°C) in separate sterile plastic bags to the laboratory for analysis. Within 30 h of sampling each sponge sample was mixed using a stomacher for 30 s before the liquid (≈5–10 ml) from each sponge was squeezed into 15 ml tubes. Each sample was diluted in peptone saline (PS, 1 g/L peptone, 8.5 g/L sodium chloride), and volumes of 100 μl from suitable dilutions were spread on Plate Count Agar (PCA) and incubated aerobically for 6 days at 15°C for determination of the aerobic plate count (APC). The remaining sample (>5 ml) was frozen at −80°C and used for extraction of DNA. After incubation, random colonies (*n* > 15) were selected from PCA plates for each drain sample using a template while ensuring all different colony morphologies were included. Colonies were re-streaked on tryptic soy agar (TSA, Merck, Darmstadt, Germany) plates, and pure colonies were stored at −80°C in 20% (v/v) glycerol peptone medium (TS/80, Technical Service Consultants Ltd., Heywood, UK) and used for identification (Section 2.2).

**Table 1:** Overview of drain samples from seafood and dairy production environments.

Drain ID	Food type	Year	Category	Drain placement
1	Fish roe	2022	Seafood	Raw roe mixing room
3	Fish roe	2022	Seafood	Raw roe glass filling
4	Fish roe	2022	Seafood	Ingredients mixing room
6	Fish roe	2022	Seafood	Raw fish roe separation
7	Shrimp	2022	Seafood	Shrimps on convoy belts (High-risk area)
8	Shrimp	2022	Seafood	Mixing of brine to shrimps (High-risk area)
9	Shrimp	2022	Seafood	Small shrimp unpacking (Inlet to high-risk area)
10	Shrimp	2022	Seafood	Big shrimp unpacking (Inlet to high-risk area)
11	Cheese	2021	Dairy	Milk pasteurization room
12	Cheese	2021	Dairy	Cheese production area
13	Cheese	2021	Dairy	Smearing room
14	Cheese	2022	Dairy	Milk pasteurization room
15	Cheese	2022	Dairy	Cheese production area
16	Cheese	2022	Dairy	Smearing room

### 2.2 Culturomics characterization of the viable microbiota in floor drains

A total of 213 drain isolates were sought and identified using matrix-assisted laser desorption ionization—time-of-flight mass spectrometry (MALDI-TOF). For MALDI-TOF identification, protein extraction was performed using fresh colonies from TSA plates (2 days at 25°C) following the ethanol/formic acid/acetonitrile protocol from Nonnemann et al. (2019), as described by Bizzini et al. (2011). Isolates were analyzed in triplicates on a Sirius Biotyper (Bruker Daltonics, Bremen, Germany) where log scores >1.7 provided genus identification, whereas identification results below <1.7 were rejected. Unidentified isolates were further analyzed using Sanger sequencing of partial 16S rRNA genes. Isolate DNA was obtained using the boiling lysis method where a fresh colony from TSA plates was resuspended in 1 ml of PS before centrifugation at 9.900 × *g* for 1 min. The supernatant was discarded and pellet resuspended in 200 μl of nuclease-free water and boiled for 10 min before centrifugation at 9.900 × *g* for 5 min after which the supernatant with DNA was transferred to new tubes. Each 16S rRNA PCR (50 μl) contained the following: 17 μl of nuclease-free water, 25 μl of DreamTaq™ PCR Master Mix, 2 μl of 10 μM forward primer (27F: AGAGTTTGATCCTGGCTCAG), 2 μl of 10 μM reverse primer (1492F: GGTTACCTTGTTACGACTT), and 4 μl of supernatant (DNA). The PCR was composed of an initial pre-denaturation step at 95°C for 10 min followed by 35 cycles of denaturation at 95°C for 30 s, annealing at 51°C for 60 s, and elongation at 72°C for 90 s followed by a final elongation at 72°C for 7 min. PCR products were purified using the ExoSAP-IT™ PCR Product Cleanup Reagent (Applied Biosystems, Waltham, Massachusetts, USA) following the manufacturer’s protocol. Aliquots of 5 μl of purified PCR product and 5 μl of primer (1492R, 5 μM) were sent for Sanger sequencing at Eurofins Genomics (Konstanz, Germany). The results from the sequencing were blasted using Nucleotide BLAST at NCBI^1^ to determine the genus of each isolate. The culturomics results from identification using MALDI-TOF and 16S rRNA sequencing were combined to determine the abundance of each genus in the drains of the seafood and dairy food productions.

### 2.3 Determination of isolates’ minimum inhibitory concentrations (MIC) toward biocides

A subset of drain isolates (*n* = 73), representing 31 genera and the diversity of the viable drain microbiota (Section 2.2), were tested for their MIC to benzalkonium chloride (BC, 500 g/L, Thermo Fisher, Kandel, Germany), peracetic acid (PAA, peroxyacetic acid, 35% w/v, Acros Organics BV, Geel, Belgium), sodium hypochlorite (SH, 10% w/v, Scharlau, Barcelona, Spain), and ethanol (ET, 99.8% v/v, Acros Organics BV, Geel, Belgium). The protocol is described in detail in Kragh et al., 2024. In short, biocides were 2-fold diluted in 10 times diluted (×0.1) Tryptic Soy Broth (TSB, Merck) as dilutant and inoculated using standardized cell suspension based on dilutions of cell cultures grown 3 days at 15°C in ×0.1 TSB. The test was conducted in a volume of 200 μl in 96-well polystyrene plates (*In Vitro*, Kratbjerg, Denmark) with an initial cell concentration of ≈2.5 × 10^5^ CFU/ml and biocides in concentration ranges observed in Supplementary Figure S1. The plates were sealed and incubated for 72 h at 15°C. Growth was measured at an absorbance of 620 nm (Multiskan FC, Thermo Fisher Scientific, Roskilde, Denmark), with a no-growth cutoff defined as absorbance readings below two times the absorbance of the negative control (0.038). The MIC was defined as the lowest biocide concentration with no growth. MIC values were tested in triplicates in two biologically independent experiments (*n* = 6).

### 2.4 Metagenomic sequencing and analysis of drain swaps

Forty-two drain samples were thawed with triplicate samples from each drain being pooled (*n* = 14) for DNA extraction. Briefly, each pooled drain sample of ≈15 ml was centrifuged at 7.400 × *g* to obtain a pellet, which was resuspended in 200 μl of the remaining liquid in the tube and transferred to a DNeasy PowerBead tube from the Qiagen DNeasy PowerSoil Kit (Qiagen, Hilden, Germany). DNA extraction was carried out following the manufacturer’s protocol. Extracted DNA was quantified using Qubit 3.0 (Invitrogen, Carlsbad, CA, United States) with the Qubit DNA HS Assay Kit (Invitrogen). Due to low DNA concentrations (median = 0.6 ng/μl), DNA samples were whole genome amplified (WGA) using the REPLI-g UltraFast Mini Kit (Qiagen) following the manufacturer’s protocol, which relies on the Phi29 polymerase to obtain enough DNA to allow shotgun metagenomic sequencing. DNA purity was assessed using the NP80 NanoPhotometer (Implen, Westlake Village, CA, USA). Amplified DNA samples of >200 ng with concentrations of >2 ng/μl were sent on ice to Eurofins Genomics for Illumina 150-bp paired-end sequencing. The raw metagenomics data were submitted to the NCBI Sequence Read Archive under BioProject: PRJNA1194757 (Supplementary Table S11). The raw sequencing data of the 14 drain samples were initially processed with CLC Genomics Workbench 23.0.4 (Qiagen, Aarhus, Denmark) to trim away adapters and low-quality reads with quality scores below 0.05. After trimming, 62.6–136.2 million reads remained for each sample with an average of 101.2 million reads. Taxonomic profiling of the filtered reads was performed with the CLC Microbial Genomics Module 23.0.2 (Qiagen) using the Qiagen Microbial Insights–Prokaryotic Taxonomy Genus Database (QMI-PTDB) as a reference. This database is curated and based on genome sequences and annotations from the NCBI Reference Sequence Database (NCBI RefSeq) with annotations from the Genome Taxonomy Database (GTDB). Taxonomic classification results were filtered to remove non-complete taxonomic classifications, and additional sequencing and WGA noise were removed by removing low-abundance species classifications with less than 0.1% abundance combined across all 14 drain samples. The filtered taxonomic abundance tables were merged and used as input for calculations of Chao1 bias-corrected alpha-diversity and beta-diversity using Bray–Curtis distance matrices in the CLC Microbial Genomics Module. Beta-diversity PCoA plots from CLC were enhanced visually to improve dot, text, and label size with Inkscape.^2^ Permutational multivariate analysis of variance (PERMANOVA, 9999 permutations) using abundance tables was applied to check for significant differences in the beta-diversity between the drains from the different processing environments (dairy, shrimp, and fish roe). Antibiotic and biocide resistance genes in the drains were identified using ShortBRED (Kaminski et al., 2015), which quantifies the abundance of resistance genes in the metagenomes by identifying unique peptide marker sequences in resistance genes and mapping reads to only those markers. The Qiagen Microbial Insight–Antimicrobial Resistance (QMI-AR) Peptide Marker Database was used for ShortBRED blast, identification, and quantification as this is curated with peptide markers from all four major databases [CARD (Alcock et al., 2020), ARG-ANNOT (Gupta et al., 2014), NCBI Bacterial Antimicrobial Resistance Reference Gene Database (Feldgarden et al., 2021), and ResFinder (Bortolaia et al., 2020)]. Resistance genes were included in the results if identity was >95%, alignment length > 95%, and read length ≥ 90 bp.

### 2.5 Whole genome sequencing and analysis of drain isolates

Thirty-one isolates (Table 2) were selected for whole genome sequencing (WGS) based on: (1) high abundance in the metagenomic drain data, (2) abundance in isolation in the viable microbiota of the drains, and (3) relevance in floor drains based on existing reviews of food processing microbiota. Isolates were grown for 2 days in 4 ml of TSB at room temperature (~20°C) before centrifugation at 7.400 × *g* to obtain a pellet, which was resuspended in 200 μl of the remaining liquid in the tube and subsequently used for DNA extraction using the Qiagen DNeasy PowerSoil Kit (Qiagen) following the manufacturer’s protocol. DNA from the 31 drain isolates was subjected to WGS by Illumina 150-bp paired-end sequencing (Eurofins Genomics). The raw sequencing data were processed with CLC Genomics Workbench 23.0.4 (Qiagen) including quality control and trimming of the sequencing reads before *de novo* assembly using default parameters. Assemblies were used for *in silico* species identification using KmerFinder v2.0 (Hasman et al., 2014; Larsen et al., 2014; Clausen et al., 2018) and the Microbial Genomes Atlas (MiGA; Rodriguez-R et al., 2018). The *L. monocytogenes* isolate were *in silico* multilocus sequence typed (MLST) using BIGSdb-Lm (Moura et al., 2016). Assemblies of all drain isolates were analyzed using ResFinder 4.1 (Bortolaia et al., 2020) to identify resistance genes and for the presence of virulence and stress genes using VFanalyzer with the reference database for virulence factors of pathogenic bacteria (VFDB, Liu et al., 2019) using default parameters and a cutoff threshold of 90% identity and coverage. Assemblies were automatically annotated using Bakta Web (Schwengers et al., 2021) with the bacterial translation table and manually explored for genetic determinants implicated in biocide tolerance. Finally, the PLSDB web tool (Galata et al., 2019; Schmartz et al., 2022), which is a comprehensive database including 34,513 (October 2023) bacterial plasmids, was used for a large-scale comparative analysis to detect the presence of plasmids in the drain isolates. The PLSDB database was used with the Mash search setting (Ondov et al., 2016) with a default maximal *p*-value of 0.1 and a minimum identity of 0.99. Any plasmid identification and additional identification of mobile elements were validated with results from MobileElementFinder (Johansson et al., 2021). The raw WGS data of the drain isolates were submitted to the NCBI Sequence Read Archive under BioProject: PRJNA1194757 (Supplementary Table S11).

**Table 2:** List of drain biofilm model isolates selected to represent the core viable microbiota and metagenome of floor drains in seafood and dairy processing environments.

Biofilm drain model isolates	GMR.	Source	Mobile genetic elements	Resistance genes
*Pseudomonas anguilliseptica C97*	1	Seafood	Tn5501	
*Pseudomonas chengduensis T33*	1	Dairy		
*Pseudomonas cremoris C118*	1	Seafood	Tn5501 + Multiple IS	
*Pseudomonas fluorescens T31*	1	Dairy	ISPpu14	
*Pseudomonas migulae C88*	1	Seafood	Tn5501 + Multiple IS	*aph(3″)-Ib*
*Brevundimonas vesicularis T3*	2	Dairy		
*Brevundimonas vesicularis C164*	2	Seafood		
*Acinetobacter guillouiae C42*	3	Seafood		*aph(3′)-VI, bla*_OXA-274_
*Acinetobacter johnsonii T53*	3	Dairy		*bla*_OXA-334_
*Moraxella osloensis T34*	5	Dairy		
*Aeromonas media T12*	6	Dairy	pfekpn2511-4	*bla*_OXA-427_ *& bla*_CMY-8b_
*Psychrobacter alimentarius C147*	7	Seafood	pVB11737_6	
*Yersinia aldovae C62*	9	Seafood	MITEYpe1, IS5075	
*Stenotrophomonas rhizophila C2*	12	Seafood		
*Kocuria rhizophila C38*	23	Seafood		
*Pseudoalteromonas translucida LU10*	32	Shrimp		
*Serratia marcescens T40*	35	Dairy	pSM22	*aac(6′)-Ic blaSRT-2, OqxB, tet(41)*
*Citrobacter portucalensis* T5	36	Dairy	pKPC-CAV1321	*qnrB9, bla*_CMY-34_ *, sugE*
*Rothia amarae* T2	40	Dairy	Multiple IS	
*Pseudoclavibacter helvolus* C31	48	Seafood		
*Chryseobacterium haifense* T23	49	Dairy		
*Chryseobacterium scophthalmum* C1	49	Seafood		
*Flavobacterium frigoritolerans* C105	52	Seafood		
*Microbacterium liquefaciens* C25	58	Seafood		
*Leucobacter luti* T63	67	Dairy	Multiple IS	
*Carnobacterium iners* MU23	94	Shrimp		
*Sphingobacterium faecium* C58	112	Seafood		
*Myroides odoratus* T56B	220	Dairy		*bla*_TUS-1_
*Rhodococcus qingshengii* C133	225	Seafood	pR85A	
*Listeria monocytogenes* C16 (ST121)	(−)	Seafood	plm5578, Tn6188	*fosX qacH*
*Shewanella oncorhynchi* C102	(−)	Seafood	Multiple IS	*mcr-4.3, bla*_OXA-549_

The isolates were selected based on (1) high abundance in the metagenomic drain data, (2) abundance in the viable microbiota of the drains, and (3) relevance in floor drains based on existing reviews of food processing microbiota. GMR: Genus metagenomic ranking based on their relative abundance among the 274 genera identified in the drains (Supplementary Table S4). (−) Genera detected but below filtering limit. A full list of identified mobile genetic elements, including insertions sequences (IS), can be seen in Supplementary Table S9.

### 2.6 Creation and repeated biocide treatment of a drain biofilm model

The 31 sequenced isolates were selected for the drain biofilm model based on the metagenomic drain abundance levels, the frequency in the viable drain microbiota, and their relevance to mimic the drain microbiota. The drain biofilm model was created on food-grade stainless steel coupons (SS coupons, 0.5 × 0.5 cm, thickness of 1 mm, AISI 316, type 4 finish). The SS coupons were washed and prepared as previously described (Kragh et al., 2020). The isolates were cultured on TSA plates for 1 to 3 days (~20°C) from stocks stored in 20% (v/v) glycerol peptone medium (TS/80, Technical Service Consultants Ltd., Heywood, UK) at −80°C. Three fresh colonies were used to inoculate each isolate individually in 4 ml of TSB with incubation for 2 days at 15°C. Equal volumes (100 μl) of each isolate were mixed and diluted 3,000× with TSB resulting in a final concentration of 10^6^ CFU/ml. Aliquots of 500 μl of this inoculation mix were used to cover SS coupons placed in wells of a 48-well plate (BioLite 48 Well Multidish F-well, Thermo Fisher Scientific, Roskilde, Denmark). Biofilms were allowed to form during incubation for 3 days at 15°C with lids on before biocide treatments commenced. Working concentrations of biocides, i.e., BC (250, 1,000, and 2,000 mg/L), SH (1,000, 6,000, and 12,000 mg/L), and PAA (500, 2,500, and 5,000 mg/L), were made by dilution of freshly prepared stock solutions using sterile water. Each biocide was used to disinfect two SS coupons (*n* = 2) with one of the three concentrations indicated above. In addition, two SS coupons (*n* = 2) were treated with sterile water as control. Before the application of biocide or sterile water, planktonic cells in suspension (~500 μl) surrounding the SS coupons were carefully removed by slow pipetting; 600 μl of biocide or sterile water was added to the SS coupons with an exposure time of 5 min following removal and 3× washing of both treated and non-treated (controls) SS coupons with 700 μl of PS. Following washing, each SS coupon was transferred carefully to microcentrifuge tubes with 1,000 μl of PS. Sessile biofilm cells were released using sonication with 50/60 kHz in a 1,000 W sonication bath (Elmasonic S 120, Thermo Fisher Scientific) for 5 min to allow for enumeration of surviving biofilm cell concentrations and detect survival of *L. monocytogenes*. Enumeration of cell concentrations was assessed by 10-fold dilutions of samples in PS with spread plating (100 μl) on TSA, counting after 4-day incubation at 15°C, and expressing as log CFU/cm^2^ for biofilm samples. Survival of *L. monocytogenes* in the biofilm was assessed by inoculation of 10-fold diluted sample volumes of 100 μl in Half-Fraser broth (48 h, 37°C, Oxoid, Thermo Fisher) before plating on PALCAM agar (48 h, 37°C, Oxoid, Thermo Fisher). The limit of detection for survival of *L. monocytogenes* was 10 CFU/SS coupons.

To allow repeated biocide treatments to occur on day 6, all biocide treatments on day 3 were done on a total of six SS coupons of which two were sacrificed for day 3 analyses. The four remaining SS coupons were allowed to regrow and form new biofilms by adding 500 μl of fresh TSB to each well after the last washing step followed by incubation for 3 days at 15°C. For each of the regrown biofilms, two SS coupons were treated with water to assess the regrown biofilm cell concentration, whereas the two other SS coupons were treated with the same biocide concentration as in the day 3 treatment.

All biocide treatments and controls were done in duplicates with the entire experiment repeated two times. To determine the efficacy of applying the same biocide concentration on initial and regrown biofilms, the sessile biofilm cell concentrations were used to calculate the log reductions (log CFU/cm^2^) after the first (day 3) and second (day 6) treatments.

### 2.7 MALDI-TOF identification of the surviving drain microbiota

To determine which bacteria survived the biocide treatments, MALDI-TOF was used for a growth-dependent identification of surviving bacteria as described above (Section 2.2). Each of the drain biofilm model isolates (Table 2) was manually added to a local MALDI-TOF database for improved genus and species identification based on three independent spectra. For assessment of the identity of the most abundant genera of surviving biofilm cells following biocide treatments (Section 2.6), five isolates were picked randomly using a template on top of the TSA plates where the most diluted biofilm samples had been spread following treatment with the highest biocide concentration (BC = 2.000 mg/L, SH = 12.000 mg/L, and PAA =5,000 mg/L). As each treatment was repeated four times (duplicates in two independent trials) on each sampling day, this resulted in the isolation of 20 colonies from each treatment (Control (sterile water), PAA, BC, and SH) on days 3 and 6, respectively, totaling 160 colonies for MALDI-TOF identification using the local custom database and MALDI-TOF scores as described above for species or genus identification.

### 2.8 Culture-independent analysis of the regrown biofilm

The remaining liquid (800 μl) from the sonicated samples (Section 2.6) containing the sessile biofilm cells was used as input for 16S rRNA amplicon sequencing to determine the composition of the microbiota of the regrown biofilms after the biocide treatments (BC, PAA, and SH at three concentrations each) and controls (sterile water after 3 and 6 days for the initial and regrown control biofilms, respectively). DNA was harvested by centrifuging the 800 μl (9.900 × *g*) and resuspending the pellet in 200 μl of the remaining liquid in the tube for DNA extraction (Qiagen DNeasy PowerSoil Kit). All biological duplicates from both biological independent experiments (*n* = 4) were used for individual DNA extractions. The V3–V4 16S rRNA region of the sample DNA was amplified and sequenced using Illumina MiSeq 300-bp paired-end sequencing (Eurofins Genomics). The quality control and trimming of the raw sequencing data of the biofilm samples were processed with CLC Genomics Workbench 23.0.4 (Qiagen) as described above. After trimming, 106.2–204.1 thousand reads remained for each sample with an average of 105.4 thousand reads. Taxonomic profiling of the filtered reads was performed with the CLC Microbial Genomics Module 23.0.2 (Qiagen) using The SILVA ribosomal RNA database (99%) reference database (Glöckner et al., 2017) for operational taxonomic unit (OTU) clustering. Taxonomic classification results were filtered to remove non-complete taxonomic classifications and sequencing noise by removal of OTUs with less than 1,000 reads across all samples. Taxonomic classification was further manually curated after filtration to merge identical taxonomic groups. This was done for *Stenotrophomonas*, *Yersinia*, *Pseudomonas*, and *Aeromonas* (Supplementary Table S8). In addition, sequences from non-complete taxonomic classifications were blasted using blastn (See footnote 1) before merging if more than one group of bacteria belonged to the same taxa. This was done for an incomplete classification of *Enterobacteriaceae* and *Micrococcaceae*, whose sequences were identified as the genera of *Yersinia* and *Rothia*, respectively. The final relative abundances were visualized as a heatmap using GraphPad Prism 10 (GraphPad Software, Boston, MA, USA). Principal component analysis was performed in GraphPad Prism 10 (GraphPad Software) to identify the effect of biocides on the microbiome composition using the relative abundance levels of each genus in samples. PERMANOVAs were conducted in CLC Microbial Genomics Module 23.0.2 (Qiagen) using the relative abundance tables to check for significant differences in the beta-diversity between the different treatments of the biofilm. Differential abundance analysis was also conducted using the sterile water treatment (day 6) as a control to compare the fold changes caused by the abundance of each genus of isolates by the different biocide treatments. The fold changes were expressed as the mean of the log_2_ fold change (log_2_ FC) for each biocide.

### 2.9 Statistical analysis and data visualization

The Student’s *t*-test was carried out using Microsoft Excel (Version 2,307) at a significance level of *p* < 0.05 to compare log-transformed microbial counts (log CFU/cm^2^). This was conducted for the cell concentrations of the drain swaps to test for differences between food production types. In addition, *t*-tests were conducted for samples of the biofilm model to compare the cell concentrations and log reductions obtained after the first and second biocide treatments.

Metagenomic and 16S rRNA amplicon sequencing data analysis and statistical comparisons were done in CLC Genomics using the Microbial Genomics Module 23.0.2 (Qiagen) with significance evaluated using Bonferroni-corrected *p*-values (*P_corrected_*) to correct for multiple comparisons and visualized in GraphPad Prism 10.

## 3 Results

### 3.1 The viable microbiota of floor drains

**Figure 2:**
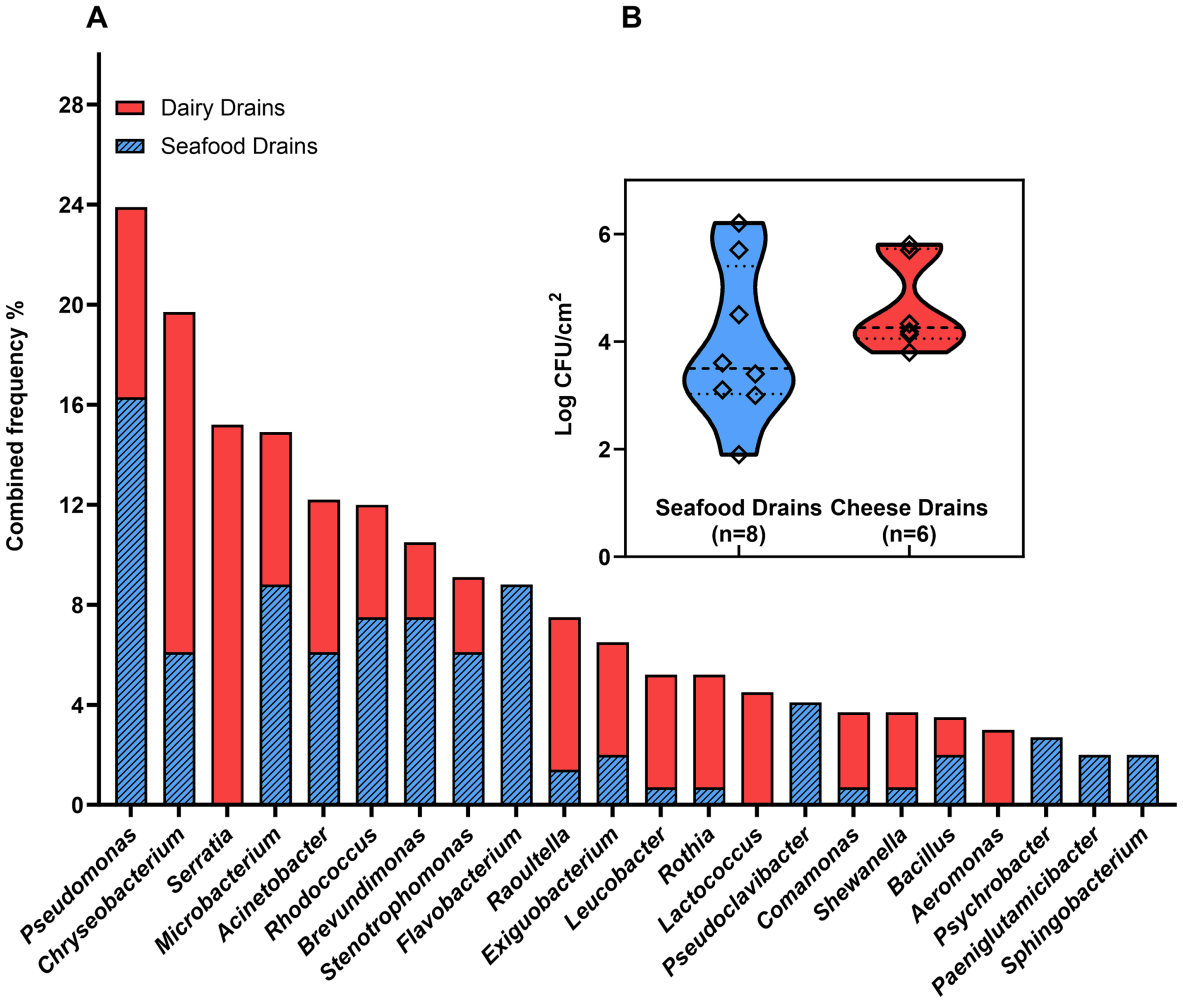
Diversity of the viable microbiota of floor drains: **(A)** Frequency of isolation of specific genera as determined by identification by MALDI-TOF and 16S rRNA Sanger sequencing; **(B)** the aerobic plate counts in floor drain samples. Drain isolates and drain cell concentrations were obtained by plating samples on TSA plates (6 d at 15°C).

Samples from 14 floor drains in seafood (fish roe and shrimp) and dairy (cheese) processing facilities were used for a culturomics characterization of the microbiota in the floor drains. Forty different genera were identified from the drains based on genus identification of 213 bacterial isolates with 22 genera having an isolation frequency > 2% (Figure 2A, Supplementary Table S1). A comparison of the microbiota of seafood drains and dairy drains found 14 genera to be present in both environments (Figure 2A). The viable microbiota of seafood drains was dominated by *Pseudomonas* (16%) followed by *Flavobacterium* (9%), *Microbacterium* (9%), *Brevundimonas* (8%), and *Rhodococcus* (8%). For dairy drains, *Serratia* (15%) dominated the drains, together with *Chryseobacterium* (14%) and *Pseudomonas* (8%). Cumulative frequencies across the FPEs showed *Pseudomonas* (24%), *Chryseobacterium* (20%), *Serratia* (15%), *Microbacterium* (15%), *Acinetobacter* (12%), *Rhodococcus* (12%), and *Brevundimonas* (12%) to be highly present, however, with *Serratia* solely isolated from dairy drains (Figure 2A). Two isolates of *Listeria* were isolated from drains in the seafood processing environment producing pasteurized fish roe. The mean aerobic plate count of the seafood drains was 3.9 ± 1.4 log CFU/cm^2^ with no significant (*p* > 0.05) difference to concentrations of dairy drains of 4.7 ± 0.8 log CFU/cm^2^. The APC ranged among the 14 drains from 2 to 6 log CFU/cm^2^ (Figure 2B).

Testing of the general biocide tolerance level in the viable drain microbiome as represented by 87 isolates belonging to 31 genera showed variability in tolerance toward commonly used biocides. This was especially clear for BC where a > 30-fold diversity in MIC was observed with 18 isolates with MICs above 5 mg/L of which seven isolates recorded MIC values ≥20 mg/L (Supplementary Figure S1, Supplementary Table S2). For SH, PAA, and ET, more than half of the isolates had identical MIC values with ≤8-fold differences in tolerance between the least and most sensitive.

### 3.2 Taxonomic profiling of the core metagenome of drains across food production types

**Figure 3:**
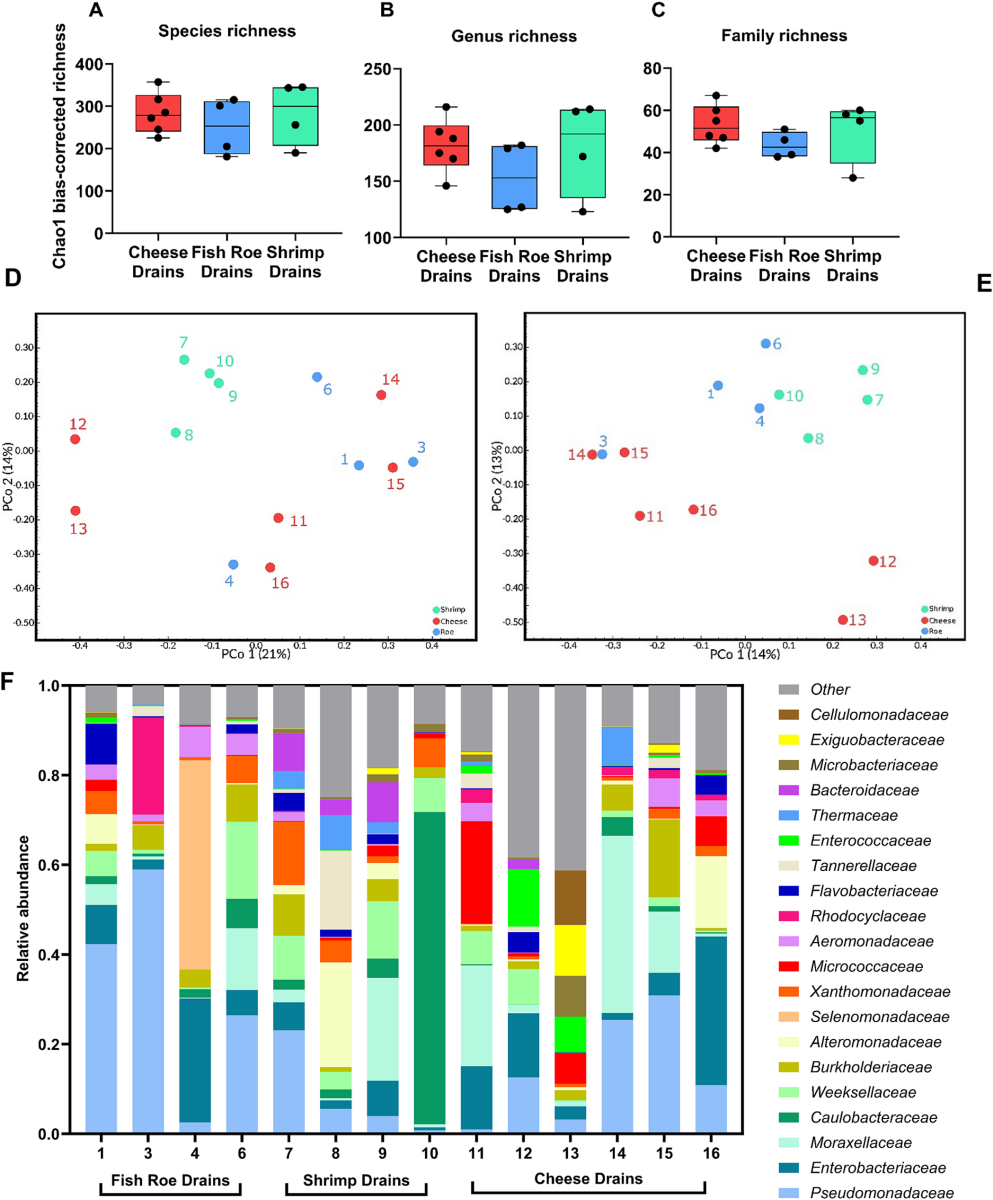
Microbial diversity and relative abundance of drain metagenomes in 14 drains from fish roe- (*n* = 4), shrimp- (*n* = 4), and cheese-processing (*n* = 6) environments. Comparison of the alpha-diversity based on Chao1 bias-corrected richness at **(A)** species, **(B)** genus, and **(C)** family taxonomic level. Beta-diversity based on Bray–Curtis dissimilarity on **(D)** genus- and **(E)** family-based taxonomy. **(F)** Relative abundance of families in the 14 drains, with the three most abundant families included from each drain, resulting in 20 families included.

Shotgun metagenomic sequencing of the 14 drains produced 62.6–136.2 million paired-end high-quality reads per sample with an average of 101.2 million reads. A high proportion of reads could not be classified to any taxa, whereas an average of 24.2 million paired-end reads were assigned to the domain of Bacteria. After filtering and removal of extremely low abundant species, a total of 665 species, 274 genera, and 89 families were identified across the drains. The differences in alpha-diversity based on Chao1 bias-corrected richness between the food production environments were not significant (Kruskal–Wallis *p* > 0.05) at species, genus, or family levels (Figures 3A–C). The beta-diversity based on Bray–Curtis dissimilarity did not clearly separate the drain metagenomes at the family or genus level (Figures 3D,E). At the genus level, the taxonomical abundances caused significant differences in the metagenome between shrimp and cheese drains (*P_corrected_* < 0.05; Figure 3D, Supplementary Table S4). The beta-diversity at the family level was in contrast not significantly different among drains from the three production types (*P_corrected_* > 0.05; Figure 3E, Supplementary Table S3). When grouped as either dairy or seafood production drains the difference in the metagenomes was significant (*P_corrected_* < 0.05) at the genus level. At both genus and family levels, the variation and complexity of the drain metagenomes were, however, apparent from the low PCoAs (Figures 3D,E). This was additionally observed when the most abundant families from each drain were evaluated based on relative abundance (Figure 3F, Supplementary Table S3), as differences among some drains were obvious with, e.g., drain 4 and drain 10 dominated by *Selenomonadaceae* and *Caulobacteraceae*, respectively, which were not dominating taxa elsewhere. Common for most drains was the high abundance of *Pseudomonadaceae* with an average of 17% ± 17% across all drains resulting in this family being one of the three dominating families in 8 of 14 drains along with *Enterobacteriaceae* (9% ± 9%) and *Moraxellaceae* (9% ± 11%; Supplementary Table S3). Common for all drains was that despite the high diversity and complexity more than half of the total drain metagenome across all three processing environments could be summarized from the abundance of the six most abundant families which contain Gram-negative species (*Pseudomonadaceae*. *Enterobacteriaceae*, *Moraxellaceae*, *Caulobacteraceae*, *Weeksellaceae*, and *Burkholderiaceae*), similarly the 20 most abundant families (Figure 3F) accounted for 84% of the total drain metagenome. The dominance of Gram-negative bacteria was also evident at the phylum level with *Pseudomonadota* (former *Proteobacteria*) accounting for 80, 68, and 70% of the drain metagenomes in fish roe, shrimp, and cheese drains, respectively (Supplementary Figure S3). Together with *Pseudomonadota*, *Bacteroidota*, *Actinomycetota* (formerly known as *Actinobacteria*), *Bacillota* (formerly known as *Firmicutes*), and *Deinococcota* made up >95% of the drain bacterial metagenomes.

**Figure 4:**
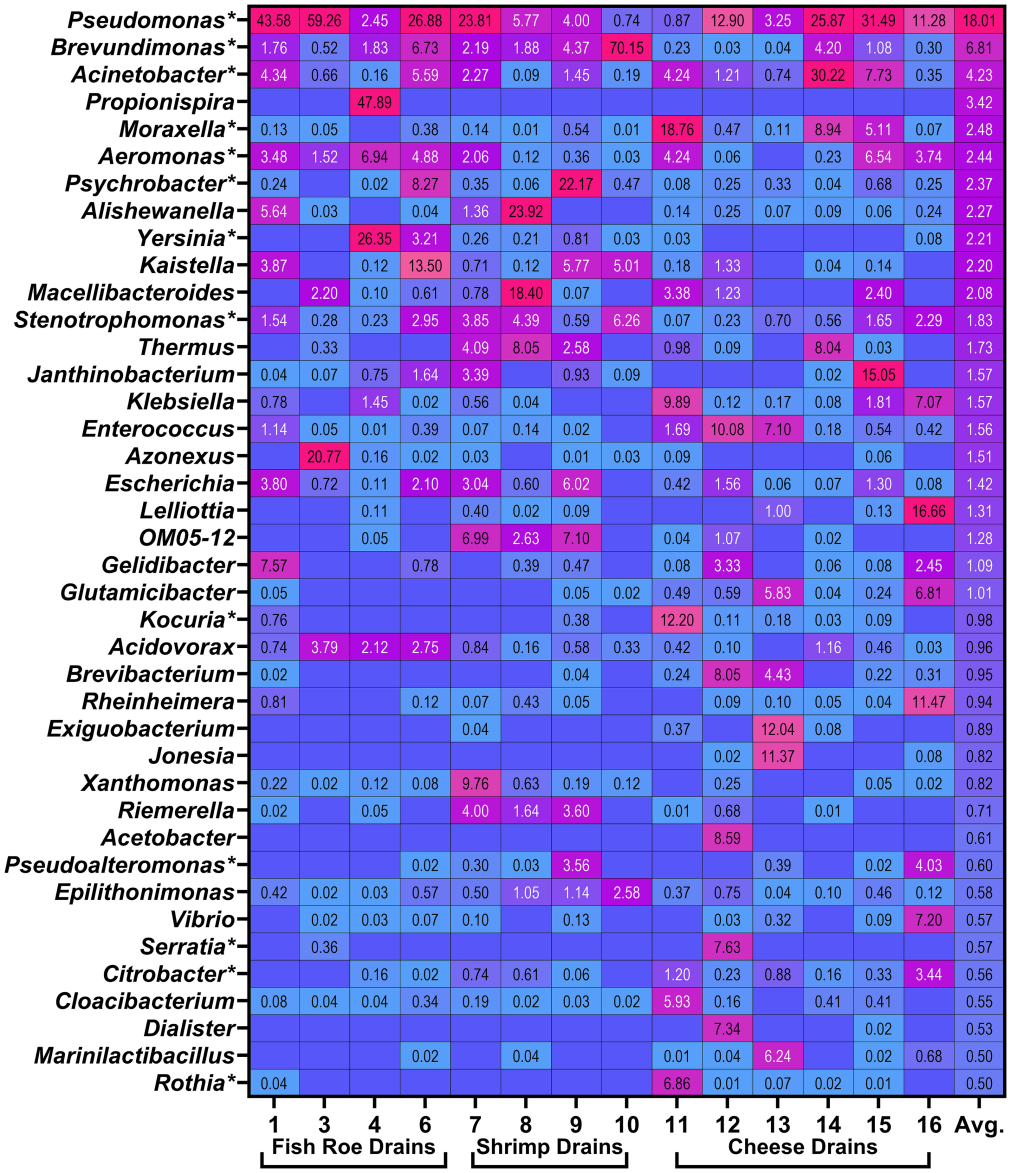
Heatmap visualizing 40 most abundant genera by highest average (Avg.) relative abundance (%) identified in the metagenomes of floor drains. Boxes without values are the result of the genera not being identified in the specific drain. Genera marked by * (*n* = 13) are included in the biofilm drain model (Section 3.4). Relative abundances for all identified 274 genera across all drains can be seen in Supplementary Table S4.

A total of 274 genera were present at combined relative abundances greater than 0.1% across all drain samples after merging identical genera and removal of incomplete taxonomic designations. Of these, a total of 131 genera were present at a cumulated relative abundance greater than 1% and 30 genera at a cumulated relative abundance greater than 10% across all drain samples (Supplementary Table S4). Of the dominating genera, 36 were present at least one time at relative abundances greater than 5%, and of these, 22 genera were on average present at a relative abundance greater than 1% (Figure 4). Interestingly, only four of these dominating genera [*Pseudomonas* (18.0%), *Brevundimonas* (6.7%), *Acinetobacter* (4.2%), and *Stenotrophomonas* (1.8%)] were present in all 14 drains with an average relative abundance as reported, while *Moraxella* (2.5%)*, Aeromonas* (2.4%)*, Psychrobacter* (2.5%)*, Enterococcus* (1.6%), and *Escherichia* (1.4%) were present in 13 drains. *Propionispira* were found in the fish roe drain (Drain 4) in the room for mixing of raw ingredients done separately from raw fish roe processing, and although abundant in that sample, it was not reflected in the overall drain metagenomes. Similarly, the dominance of *Caulobacteraceae* in shrimp drain 10 was reflected in the high abundance of *Brevundimonas*, which were, however, widely present in other drains across all three FPEs (Figure 4).

### 3.3 Prevalence of antibiotic resistance gene markers in the resistome of floor drains

**Figure 5:**
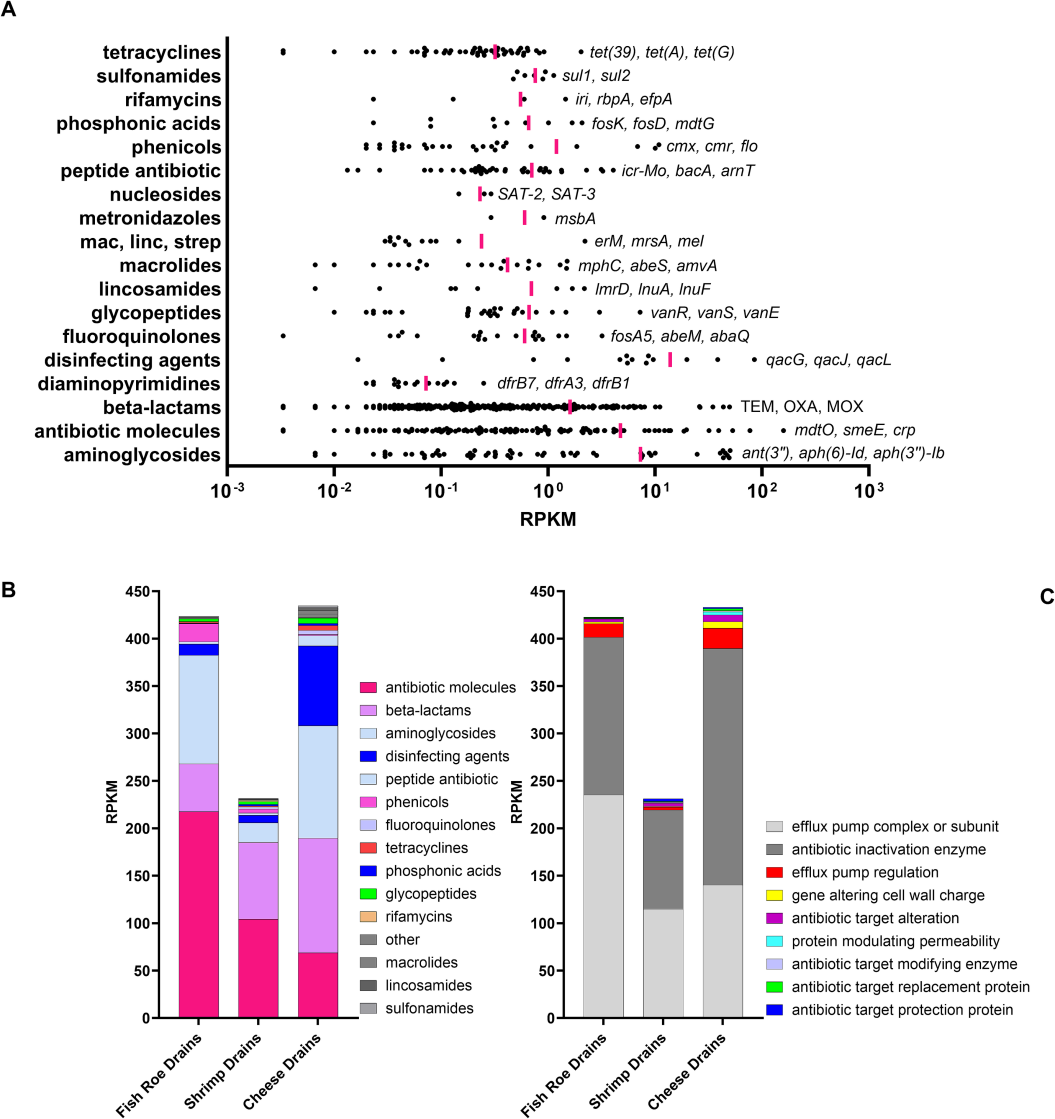
Resistome identification and normalized abundance [Reads Per Kilobase per Million mapped reads (RPKM)] of ARG markers in floor drains using ShortBred. **(A)** The abundance of ARGs (•) to specific antibiotic drug classes with vertical pink lines marking mean RPKM for all markers in each drug class. The gene names for the three most abundant ARGs are listed for each drug class. Bar graphs showing the **(B)** normalized abundance are summarized by drug class and **(C)** resistance mechanism for each drain.

A total of 699 unique antibiotic resistance gene (ARG) markers were identified using ShortBRED in the metagenome of floor drains (Supplementary Table S5). The identified genes belonged to 18 different antibiotic classes (CARD Ontology) with 259 genes linked to beta-lactam resistances as the most frequently observed ARG resistance markers (Figure 5A, Supplementary Table S5). However, the most abundant group based on normalization using Reads Per Kilobase per Million mapped reads (RPKM) were 119 markers belonging to the multi-resistance and undefined group of “antibiotic molecules” (Figure 5A). Of these 119 markers, the majority (102) and those with the highest RPKM were efflux pump complexes such as *mdtO*, *smeE*, and *mexB*. Other highly abundant efflux pumps included *qacG*, *qacJ*, and *qacL*, which are all related to quaternary ammonium compound (QAC) resistance and belong to the ARG class of “disinfecting agents and antiseptics” (Figure 5A). QAC resistance genes were identified in drains from all three FPEs but were most abundant in cheese drains (Figure 5B). Other QAC resistance genes identified included *mdfA*, *qacE*, *qacH*, and *sdrM*. In general, the efflux pump-mediated resistance mechanism dominated along with resistance markers related to antibiotic enzyme inactivation, which was driven by a high abundance of genes conferring resistance toward aminoglycosides [ant(3″), aph(6)-ld, aph(3″)-lb] and beta-lactams (*bla*_TEM_, *bla*_OXA_, *bla*_MOX_; Figure 5, Supplementary Tables S5–S7). For the overall resistome, there were no significant differences among shrimp, fish roe, and cheese drains (*P_corrected_* = 0.085). Similarly, the difference between the resistome of shrimp and fish roe drains was not significant (*P_corrected_* = 0.77). However, a much lower overall ARG marker abundance was observed in shrimp drains.

### 3.4 Selection and analysis of representative isolates for the creation of a drain biofilm model

To study how the drain microbiota is affected by repeated biocide treatments, a multi-genera biofilm drain model was created to reflect the observed microbiome diversity of the studied food industries. Therefore, based on the metagenomic abundance and isolation frequency across the food facilities five isolates of *Pseudomonas* (*P*. *cremoris*, *P. migulae*, *P. anguilliseptica*, *P. fluorescens*, and *P*. *chengduensis*) were included along with *Acinetobacter* (*A. guillouiae* and *A. johnsonii*) and two isolates of *Brevundimonas* (*B. vesicularis*). In addition, two isolates of *Chryseobacterium* (*C. scophthalmum* and *C. haifense*) were included as the second most abundant viable genera, while ranked as the 49^th^ most abundant genera in the metagenomic data (Figure 2, Supplementary Table S4). Similarly, *Microbacterium* (*M. liquefaciens*) was included due to its high frequency in the viable microbiota, while ranked 58^th^ of the metagenomically most abundant genera. Eleven more isolates (*Moraxella osloensis*, *Aeromonas media*, *Psychrobacter alimentarius*, *Yersinia aldovae*, *Stenotrophomonas rhizophila*, *Kocuria rhizophila*, *Pseudoalteromonas translucida*, *Serratia marcescens*, *Citrobacter portucalensis, Rothia amarae*, and *Pseudoclavibacter helvolus*) were included based on presence in the viable drain microbiota and a place among the 50 most abundant genera in the metagenome. *Flavobacterium* (*F. frigoritolerans*) were included as the second most viable abundant genera in seafood drains and identified in four of six metagenomes of cheese drains. Four isolates were included based on frequent drain isolation in other studies (Fox et al., 2014; Rodríguez-López et al., 2019, 2020) combined with either co-isolation from dairy and seafood drains in the present study (*Leucobacter luti, Rhodococcus qingshengii*, and *Shewanella oncorhynchi*) or high abundance in shrimps (*Carnobacterium*, Unpublished). In contrast, *Myroides*, and *Listeria* isolated from drains in the present study were included as model organisms based on their ability to be either multidrug-resistant (*Myroides* spp., Licker et al., 2018; Lorenzin et al., 2018) or persistent in food production environments (*L. monocytogenes*, Belias et al., 2022; EFSA Panel on Biological Hazards (BIOHAZ) et al., 2024). The included *L. monocytogenes* was typed as ST121. Finally, an isolate of *Sphingobacterium* (*S. faecium*) was included due to its frequency in seafood drains and based on reports of some isolates’ ability to degrade pollutants such as chlorinated pesticides and hence of interest in the context of repeated biocide treatments of a multispecies biofilm model (Abraham and Silambarasan, 2013; Satti et al., 2021).

The final 31 isolates from 24 genera collectively represented 58, 53, and 47% of the microbial diversity observed in the metagenome of fish roe, shrimp, and cheese drains, respectively (Supplementary Table S8). Similarly, 76 and 81% of the viable dairy and seafood drain microbiota are reflected by the included isolates in this model (Supplementary Table S1).

Of the 31 isolates, nine possessed ARGs mainly toward beta-lactams with *C. portucalensis, Se. marcescens, A. guillotine*, and *Sh. oncorhynchi* harboring ARGs to more than one drug class (Table 2). QAC resistance efflux pumps *sugE*, *oqxB*, and *qacH* were observed in *C. portucalensis, Se*. *marcescens*, and *L. monocytogenes*, respectively. The *qacH* found on the *L. monocytogenes* Tn6188 transposon was, together with the heat resistance gene *clpL* on plasmid *L. monocytogenes* plm5578, the only resistance gene identified on mobile genetic elements among the genomes of the 31 isolates. In addition, *P. alimentarius, Se. marcescens, R. qingshengii, C. portucalensis*, and *A. media* carried plasmids, but none contained ARGs.

### 3.5 Repeated biocide treatments of the drain biofilm model

**Figure 6:**
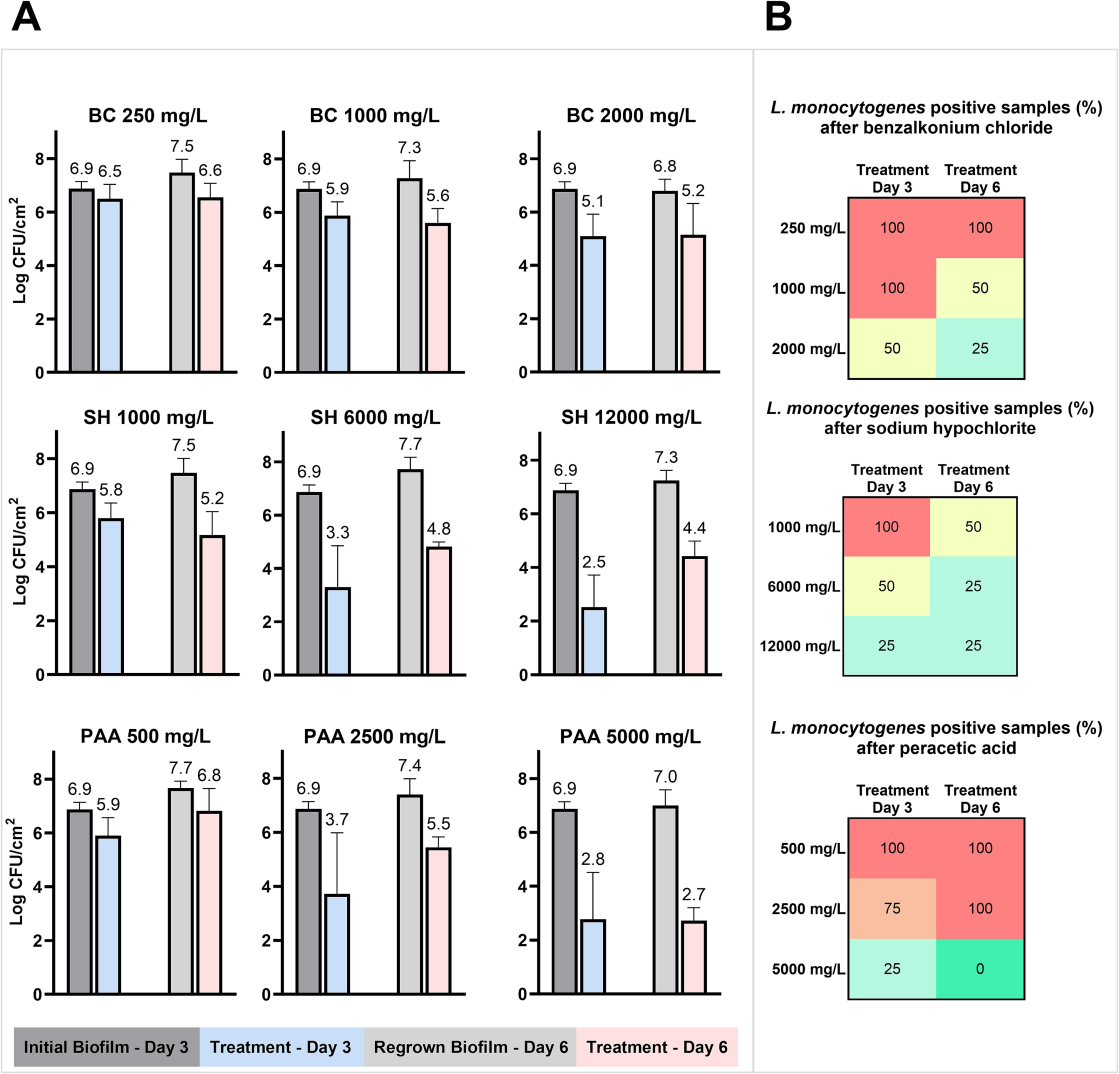
Biocide treatment of drain biofilms formed on SS coupons. **(A)** Mean cell concentrations in the biofilm (*n* = 4) before and after biocide treatment with different concentrations of biocides (BC, SH, and PAA) on days 3 and 6 with a biofilm regrowing phase between. **(B)** The percentage of *L. monocytogenes* positive drain biofilms after treatment with biocides and selective enrichment for *L. monocytogenes* after each treatment (*n* = 4).

The 31 isolates were mixed to a starting concentration of 10^6^ CFU/ml, which resulted in an initial biofilm on the SS coupon of 6.9 ± 0.3 log CFU/cm^2^ after 3 days at 15°C (Figure 6A). The suspension of planktonic cells around the SS coupons with the initial biofilm had a concentration of 9.2 ± 0.2 log CFU/ml. BC treatments at low, high, or double industrial concentrations (250, 1,000, and 2,000 mg/L) were unable to reduce the biofilm by more than 2 log CFU/cm^2^. Correspondingly, low reductions (< 2 log CFU/cm^2^) of the biofilm were also the result of treatments with low industrial concentrations of SH (1,000 mg/L) and PAA (500 mg/L). Conversely, higher concentrations of both SH (6,000–12,000 mg/L) and PAA (2,500–5,000 mg/L) reduced the biofilm by 3.4–4.5 log CFU/cm^2^ with the greatest effect achieved at the highest concentrations for both biocides (Figure 6A). After each biocide treatment, surviving biofilms regrew to cell concentrations of 7.3 ± 0.6 log CFU/cm^2^ after 3 days at 15°C, which were in all but one case higher than in the initial biofilm. For controls treated with water, the regrown biofilms reached a concentration of 7.0 ± 0.6 log CFU/cm^2^ similar to the initially formed biofilm. The cell concentrations in regrown biofilms tended to be highest for those treated with the lowest biocide concentrations, as observed in a 0.7 log CFU/cm^2^ difference between the lowest and highest concentrations of BC and PAA. No significant (*p* > 0.05) changes in log-reductions between the first and second biocide treatment were observed despite three of nine regrown biofilms showing increased tolerance to the repeated biocide treatment (6,000–12,000 mg/L SH and 2,500 mg/L PAA), while one regrown biofilm exhibited increased sensitivity to the 1,000 mg/L SH treatment (Figure 6A).

The biocide treatments only inactivated *L. monocytogenes* below the limit of detection (10 CFU/SS coupons) at the highest concentration of PAA and only after the second treatment (Figure 6B). In total, 52 of 80 treated biofilm samples (65%) were positive for *L. monocytogenes.* There was, however, no increase in the number of positive samples between the first and second treatments, as 72.5% of the samples were positive after treatment on day 3, while 57.5% were positive after treatment on day 6. In addition, increases in biocide concentrations always resulted in fewer *L. monocytogenes*-positive samples (Figure 6B).

### 3.6 Changes in the drain biofilm microbiome in regrown biofilms following biocide treatment

**Figure 7:**
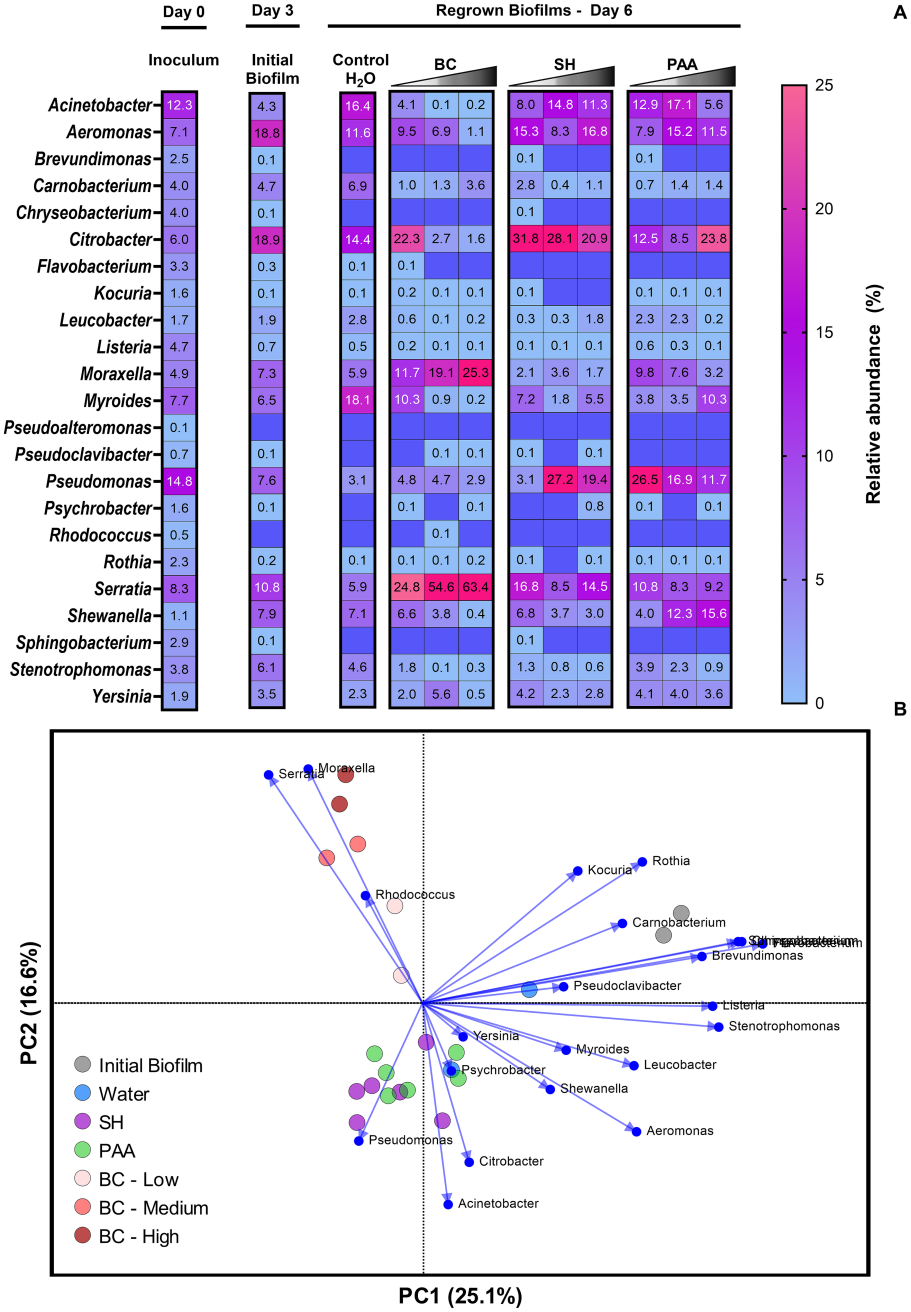
Culture-independent analysis of the drain biofilm microbiome changes after biocide treatments with each average based on 16S rRNA amplicon sequencing of biological independent replicates (*n* = 4). **(A)** Relative abundances of the microbiome in the inoculum, initial biofilm, and regrown biofilms on day 6 after treatments with either sterile water, BC, SH, or PAA on day 3. **(B)** PCA of microbiomes shown in the heatmap above and the differences in each genus caused by biocide treatments as shown by PC scores and PC loadings.

Culture-independent 16 s rRNA amplicon sequencing identified reads mapping to 23 of the 24 genera, which were in the inoculum suspensions used for biofilm startup. Reads mapping to *Microbacterium liquefaciens* were not identified. Similarly, *Pseudoalteromonas* and *Rhodococcus* were not found in the initial biofilm (day 3) formed on the SS coupons, leaving the biofilm consisting of 28 species belonging to 21 genera with low relative abundance (>2%) of 11 genera (Figure 7A). This initial biofilm was dominated by *Citrobacter* (18.9%) and *Aeromonas* (18.8%), followed by *Serratia* (10.8%), *Shewanella* (7.9%) and *Pseudomonas* (7.6%; Figure 7A, Supplementary Table S10). Following treatment with water, the diversity of the regrown biofilms (day 6) did not decrease as 12 genera had >2% in relative abundances; however, the abundances shifted to higher content of *Myroides* (18.1%) and *Acinetobacter* (16.4%) accompanied by *Citrobacter* (14.4%) and *Aeromonas* (11.6%). The impact of the three biocides on genera diversity was biggest for BC, where increasing BC concentrations reduced the number of genera with relative abundances exceeding 2% from 12 (H_2_O control) to 9, 7, and 4 for 250, 1,000, and 2,500 mg/L BC treatments, respectively. For increasing concentrations of SH and PAA, the selective pressure on genera was less pronounced as the number of genera >2% in relative abundance was reduced from 12 in the control to 10, 8, and 8 genera and 11, 11, and 8 genera for various concentrations of SH and PAA, respectively (Figure 7A). Specifically, the treatment with BC led to significant (*p* < 0.05) changes within the overall drain biofilm microbiome as *Serratia*, *Moraxella*, and *Citrobacter* made up 57% of the regrown biofilm even after low treatments with 250 mg/L BC, while *Serratia* alone constituted 55–63% of the regrown drain biofilm microbiome after treatments with higher industrial concentrations (1,000–2,000 mg/L) of BC. This change was obvious in beta-diversity analysis, where regrown biofilms treated with the highest concentrations of BC clustered far apart from regrown biofilms after sterile water, PAA, and SH treatments (Figure 7B). In the beta-diversity analysis, the initial biofilm clustered separately (Figure 7B), which could be explained by several genera, e.g., *Brevundimonas, Chryseobacterium, Pseudoclavibacter, Psychrobacter*, and *Sphingobacterium*, being detected in low abundances (≤ 0.1%), but not in the water treated control biofilm from day 6. These biofilms treated with water clustered near biofilms regrown after PAA and SH treatments (Figure 7B), which mostly contained not only *Citrobacter*, *Acinetobacter*, and *Aeromonas* but also, to a higher degree, *Pseudomonas* (Figures 7A,B). These four genera also largely explain the difference in biofilms after BC treatment with PC loadings directly opposite to BC biofilms (Figure 7B).

Interestingly, *Pseudomonas* spp., which were represented by five different species in the inoculum (14.8% relative abundance), were not observed with high abundance in either the initial biofilm (7.6%), water control (3.1%), or after BC treatments (2.9–4.8%). The overall changes in the regrown biofilm microbiome after SH or PAA treatments were not significantly different (*P*_corrected_ > 0.05) compared to when treated with water. In addition, regrown PAA- and SH-treated biofilms clustered together in the PCA (Figure 7B), irrespective of biocide concentrations, which also reflected the relative abundance increases and decreases of several genera (e.g., *Acinetobacter* and *Aeromonas* for both PAA and SH) not following the changes in biocide concentrations (Figure 7A). For *Citrobacter* and *Pseudomonas*, there was a slight decrease in their dominance with increased concentrations of SH and PAA, respectively. The relative increase in *Pseudomonas* after PAA and SH treatments and the increase in *Serratia* after BC represented the most significant (*P*_corrected_ < 0.05) changes in relative abundances when all biocide concentrations of the same biocide (*n* = 12) were compared against the water control (*n* = 4) as observed in Supplementary Figure S2. Interestingly, *L. monocytogenes* recorded its highest relative abundance in the inoculum (4.7%) and in the initial biofilm (0.7%) and were never above these percentages in any of the regrown biofilms, but detectable in all biofilms (Figure 7A).

### 3.7 Changes in the surviving viable biofilm microbiota after biocide treatments

**Figure 8:**
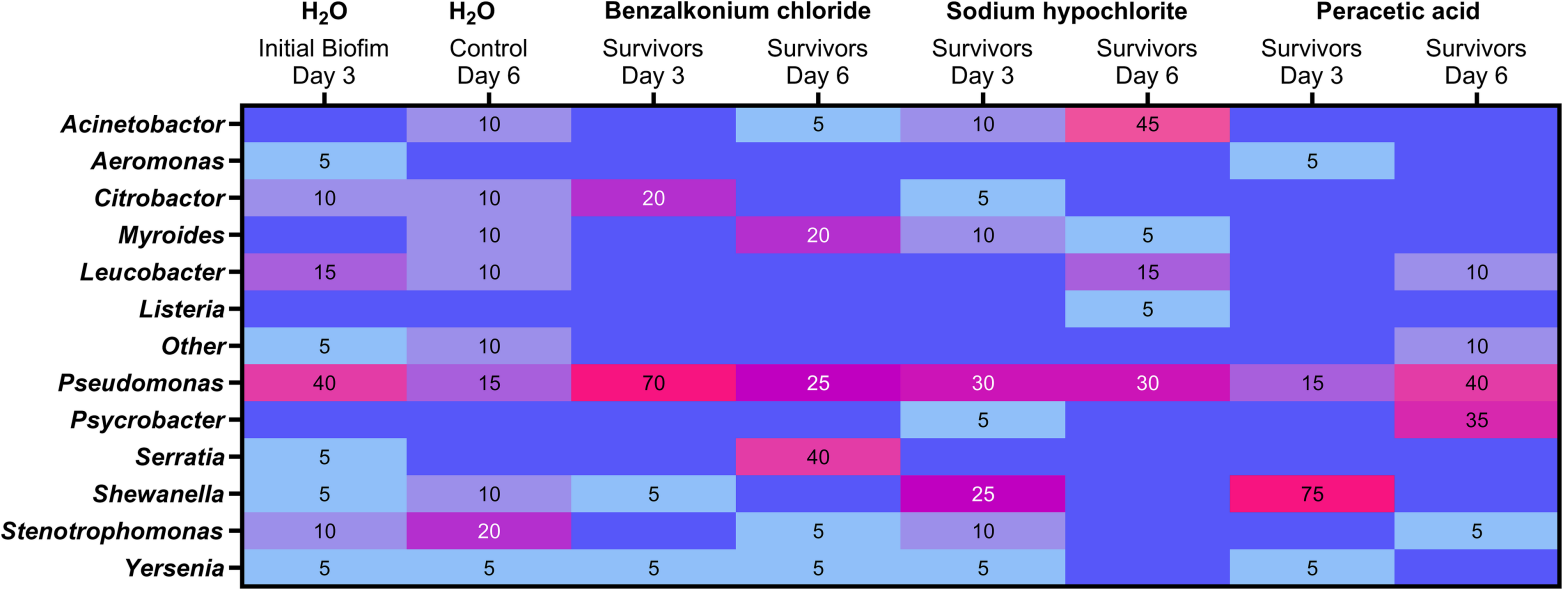
Heatmap of culture-dependent analysis using MALDI-TOF genus identification of the viable bacteria dominating in the drain biofilms after biocide treatments on days 3 and 6. Presence is given in percentage (%) and based on a random selection of colonies (*n* = 20) from each treatment (n_total_ = 220) with colonies originating from the most diluted APC, thus reflecting the most abundant genera (≥5%) in the surviving biofilms.

Multiple genera (>8) were observed among the colonies reflecting the dominating culturable and viable bacteria in the initial biofilms. *Pseudomonas* spp. accounted for 40% (*n* = 8/20) of the initial control biofilm (H_2_O, day 3) isolates identified (Figure 8). Similarly, more than eight genera were observed in the control biofilm on day 6; however, no genera dominated as *Stenotrophomonas* with 20% (*n* = 4) was the most frequently detected genera. The number of genera among the surviving bacteria decreased after biocide treatments (BC = 2.000 mg/L, SH = 12.000 mg/L, PAA =5,000 mg/L), e.g., BC treatment on day 3 led to *Pseudomonas* (*Pseudomonas migulae*) accounting for 70% (*n* = 14) of the viable biofilm isolates. *Pseudomonas* were overall the most frequently identified viable survivors after biocide treatments and were present in all surviving biofilms. Treatments on day 3 with SH left several surviving genera, including *Shewanella* (25%, *n* = 5), while PAA treatments on day 3 resulted in *Shewanella* constituting 75% (*n* = 15) of the survivors (Figure 8). *Shewanella* was, however, not observed among the dominating survivors after treatments with BC, SH, or PAA on day 6 and similarly occurred in the low abundance of 5–10% in the initial and control biofilms. Interestingly, *Serratia*, which harbors the QAC efflux *oqx*B gene, went from being present (5%, *n* = 1) in the initial biofilm and not observed among the dominant survivors after the first BC treatment on day 3 to being the dominant isolate after BC treatment on day 6 (40%). Similarly, strikingly were the 35% (*n* = 7) of *Psychrobacter* after treatment with PAA on day 6. For *Acinetobacter*, an increase was observed from 10% (*n* = 2) after the first treatment with SH on day 3 to an abundance of 45% (*n* = 9) after SH treatment on day 6. *Listeria* were in contrast only among the dominating viable colonies after SH treatment on day 6, thus reflecting it mainly being present in lower abundances of less than 5% in biofilms.

## 4 Discussion

This study aimed to define a core microbiome in drains within FPEs to create a representative biofilm model for biocide testing. Using this multispecies drain biofilm model composed of 31 bacterial isolates representing 24 genera, it was demonstrated that commonly used biocides selected for survival and regrowth of different genera after repeated exposure and that in the instance of repeated use of BC, the dominance of *Serratia*, could be explained by the carriage of a QAC efflux pump of *S. marcescens.* Below, we will discuss the characterization of the metagenome and resistome of drains and how this was used in the design of a representative floor drain biofilm model. Finally, we will discuss results from the repeated biocide treatments of drain biofilms and perspectives for the design of biocide rotation schemes to prevent the development of biocide-tolerant biofilms in the industry.

### 4.1 Floor drain microbiomes are diverse and complex but dominated by a few highly abundant taxa

Floor drains are reported to be the site with the highest microbial load and the biggest contributor to microbial diversity than other surfaces in the FPE (Cobo-Díaz et al., 2021; Xu et al., 2023). This was reflected in diversity among the 14 floor drains of this study (Figure 3) where 36 genera were present at least one time at relative abundance >5% in individual drains, while 22 of those had mean abundance levels >1% across all floor drains (Figure 4). This shared core microbiome underlines the presence of a core microbiome in different, yet microbiologically similar, wet food processing production facilities.

The results showed Gram-negative bacteria, as represented by the phylum *Pseudomonadota* dominated in both cheese, shrimp, and fish roe drains at levels of 68–80% despite the difference in food production type (Supplementary Figure S3). Similar 70–98% dominance of *Pseudomonadota* was observed in drains of three different beef-processing plants (Palanisamy et al., 2023), while 34–52% were found in metagenomes of Canadian food production facilities (Caraballo Guzmán et al., 2020). *Pseudomonadaceae* dominated at 17% in the present study and 22–26% in Palanisamy et al. (2023), with *Moraxellaceae* (>9%), being the second most abundant family in both studies together with *Enterobacteriaceae* (>9%) in the present study (Figures 3, 4). *Pseudomonas* spp., which is a genus consisting of species known to form biofilms and spoil food products, especially dominates wet FPEs as shown in a meta-study of microbiomes from surfaces in 39 different facilities (Xu et al., 2023). *Pseudomonas* spp. have been shown to rapidly populate new processing facilities for pork or salmon products, leading to >50% in the sequenced microbiome (Cobo-Díaz et al., 2021; Thomassen et al., 2023b). Interestingly, *Pseudomonas* remained the most dominant genera on cleaned and disinfected surfaces in both a salmon facility and beef industry (Thomassen et al., 2023b; Palanisamy et al., 2023), which points to insufficient removal of this genus.

An important limitation of the metagenomic analysis is the sequencing of DNA from both viable, non-culturable, and dead bacteria. Therefore, the metagenomic characterization of floor drain microbiomes was supplemented by culturomics analysis, which revealed a diverse viable microbiome leading to the isolation of 40 different genera that largely concurred with the genera dominating in metagenomic sequencing results, including high numbers of *Pseudomonas, Acinetobacter, Brevundimonas* and *Stenotrophomonas* (Figure 2). Genera common in the viable microbiome but less abundant in the metagenome included *Chryseobacterium, Serratia*, and *Microbacterium. Serratia* spp., which belongs to *Enterobacteriaceae*, were widely present in the drains (Figure 3A). *Serratia* spp. are frequently isolated in different types of food processing facilities (Møretrø and Langsrud, 2017) and are known to be among the more tolerant bacteria to cleaners and disinfectants (Langsrud et al., 2003; Boyce, 2023). *Microbacterium* spp. have been found with abundance ≥7% in metagenomes of several drains in a beef-processing facility (Palanisamy et al., 2023), while *Chryseobacterium* spp. have, in contrast, rarely been classified as part of the resident microbiome of FPEs despite being isolated from a wide range of environmental sources (Møretrø and Langsrud, 2017; Mwanza et al., 2022). The reasons for this discrepancy are not known. Interestingly, both *Microbacterium* and *Chryseobacterium* failed to establish themselves in the drain biofilm model (Figure 7A).

### 4.2 Design of a representative floor drain microbiome for testing biocide tolerance development

Thirty-one isolates were deemed sufficient to capture the core microbiota and diversity of floor drains across FPEs, as most of the dominating genera were found in all three production environments (Figures 2, 4). This broadens the applicability and results of the model to the whole food industry. As discussed above, *Pseudomonas* must be included in the model to reflect the drain microbiome (Møretrø and Langsrud, 2017) and lead to an accurate assessment of biocide efficacies on industry-like biofilms (Fagerlund et al., 2017; Charron et al., 2023; Rolon et al., 2024). With 22 genera metagenomically present at abundances of >1% in the FPEs, we deemed it necessary to include enough isolates to capture at least half of the metagenomic abundance and >75% of the viable drain microbiome (Supplementary Tables S1, S8). To compensate for the diversity and high abundance of some genera, it is reasonable to include two or more isolates from the same genera (Lake et al., 2024) and this led us to include five *Pseudomonas* isolates (Table 2). Previous biofilm experiments have shown that biocide survival is, however, not necessarily linked to the complexity of the biofilm as *L. monocytogenes* performed better in mono-species biofilm on conveyor belt coupons of polyvinyl chloride (PVC), while a dual-species biofilm with *Pseudomonas* spp. on stainless steel yielded better survival than the mono-species biofilm (Fagerlund et al., 2017; Dass et al., 2020; Thomassen et al., 2023a). The composition of the complex biofilm model will evidently lead to cooperative but also competitive interactions as some genera outgrow others. The biofilm models should not be designed to limit these, as the results of interactions can vary depending on the stress conditions applied to the model (Li et al., 2021). Specifically, Lake et al. (2024) observed how *L. monocytogenes* had initial higher survival in mono-species biofilms, but when cycles of follow-up cleaning and disinfection procedures were applied again and again, they observed better survival of *L. monocytogenes* in the multispecies biofilm which might shelter the pathogen in the long term despite competitive interactions. Importantly, our biofilm model composition led to consistent levels of *Pseudomonas* survival across the different controls and biocide treatments (Figure 8), as would be expected based on its high abundance in this (Figure 3) and other studies.

The inclusion of a pathogen of interest, e.g., *L. monocytogenes*, is based on the relevance for food safety, as they are rarely found in abundance justifying their inclusion. Similarly, the initial concentrations of isolates could be discussed as their abundance in the drains of FPEs clearly was not even (Figure 4). We chose to go with a standard preparation protocol for all isolates and mixing even volumes, which led to some isolates being present in slightly higher concentrations than others in the biofilm inoculum (Figure 7A). To further simulate the true biofilm conditions and abundances, microbial samples and nutrients taken directly from the environment can be used as inoculum, which, however, limits reproducibility for other researchers (Fernández-Gómez et al., 2022; Flores-Vargas et al., 2023). We chose to conduct the biofilm assays in undiluted laboratory media and use a temperature of 15°C to represent a worst-case soiled scenario and realistic FPE temperatures, respectively. This approach gave high and stable biofilm concentrations ≈ 7.0 log CFU/cm^2^ on the stainless steel (Figure 6A). It is common practice to apply full-strength media in biocide efficacy studies to investigate when, where, and why cleaning and disinfection programs might fail as this often occurs in hard-to-reach niches, where nutrients and dirt build up (Fagerlund et al., 2021; Rahman et al., 2022; Thomassen et al., 2023a). These niches are typically those, where mechanical brushing and cleaning fail to reach. We chose to leave out mechanical cleaning, but it should be noted that biocide manufacturers do recommend mechanical scrubbing as part of the cleaning and disinfection and, in addition, also recommend increasing biocide concentration when faced with persistent problems (Fagerlund et al., 2020).

### 4.3 Biocides alter the microbiome biofilm composition but not the biocide tolerance

In our study, the simulation of a worst-case “weekend” scenario with 3 days allowed for undisturbed biofilm formation on stainless steel, which meant that even at the highest recommended industrial concentrations or more of PAA and SH, there was a surviving biofilm microbiome of ~3–4 log CFU/cm^2^ (Figures 6A, 8). Following biocide treatments, it was observed that surviving microbiomes were altered in a manner that depended on the applied biocide, thus indicating a selective effect caused by biocides (Figure 7). Moreover, increasing biocide concentrations decreased the alpha-diversity in the biofilm that regrew after the first treatment. Reducing the diversity might not be advantageous as observations from microbiome samples from drains in factories after intense and long sanitation procedures showed that the surviving regrowing microbiomes produced a bigger biofilm mass making them more difficult to eradicate (Wang et al., 2024). We observed similarly that following PAA and SH treatments, there was an increase in overall biofilm concentration (Figure 6A) and a significant increase in *Pseudomonas* abundance (Supplementary Figure S2), whereas BC treatments caused *Serratia* and *Moraxella* to increase (Figure 7). The culture-dependent analysis of biocide-treated biofilms confirmed that *Pseudomonas* was a frequent survivor after all treatments (Figure 8), as also observed in other biofilm studies with mixes of environmental isolates from the food industry (Fagerlund et al., 2017; Lake et al., 2024). It is well known that *Pseudomonas* biofilms would potentially shelter foodborne pathogens such as *L. monocytogenes* (Thomassen et al., 2023b).

While the biocide altered the composition of the regrowing biofilm, these biofilms were not more tolerant to applications of the same biocide 3 days later (Figure 6A). However, *L. monocytogenes* prevalence decreased after the second treatment (Figure 6B), which means that at higher “industry” biocide concentrations, there is no adaptation of *L. monocytogenes* toward PAA and SH. This aligns with what has previously been shown for these biocides at lower biocide concentrations (Aarnisalo et al., 2007; Riazi and Matthews, 2011; Kastbjerg and Gram, 2012; Kragh et al., 2024). For intermediary biocide concentrations of 512 mg/L, Rahman et al. (2022) showed how repeated exposure of suspensions of *Listeria innocua* to PAA and SH led to no significant changes in log reductions, while repeated treatment with BC became less effective. The material to which the biofilms are attached also plays a role in the development of tolerance. Lake et al. (2024) reported that repeated disinfection of multispecies biofilm on PVC caused decreased reductions, an observation not found for the same biofilms on stainless steel. For multispecies and mono-species biofilms of *L. monocytogenes* formed on PVC, Fagerlund et al. (2017, 2020) similarly showed that repeated biocide treatments were less effective on day 7 than day 4. Together, this indicates that changes in biocide tolerance likely relate to the age of biofilm, biocide concentration, and the material, where more porous materials such as PVC may offer better sheltering and formation options than stainless steel.

Regarding the removal and inactivation of biofilms, PAA has previously been shown to be better at biofilm eradication than SH and BC (Cruz and Fletcher, 2012). This was also the case in the present study both quantitatively for total biofilm concentration and for *L. monocytogenes* prevalence (Figure 6). We have, in addition, previously shown how PAA was less affected by organic matter and biofilm (Kragh et al., 2024). In that regard, it is important not to only adjust the concentration of the applied biocide as we observed a very limited effect of doubling the concentration of BC and SH. These findings are in line with Barroso et al. (2019), who observed that after obtaining a 4-log reduction, a plateau was reached where further increasing biocide concentrations did not increase the inactivation. This implies that simply increasing biocide concentrations when faced with persistence problems is an inadequate solution, which, in addition, results in increased biocide expenses, equipment tear, and hazard exposure for cleaning personnel.

Benzalkonium chloride residues can be detected on surfaces after disinfection and continue to exert a selective pressure (Møretrø et al., 2017). We observed that *S. marcescens* was among the most BC-tolerant isolates with MIC ≥20 mg/L (Supplementary Table S2) and that this high MIC value could be explained by its carriage of a QAC efflux pump OqxB (Table 2). Interestingly, the included ST121 *L. monocytogenes* isolate carrying the *qacH* efflux pump gene had a markedly lower MIC value (2.5 mg/L) and low abundance in regrown biofilm after BC treatments (Figure 7). We and others have previously shown how the carriage of the major BC-tolerant genes (*qacH, brcABC, emrC*, or *emrE*) in *L. monocytogenes* does not contribute to increased tolerance at industrially relevant biocide concentrations (Møretrø et al., 2017; Barroso et al., 2019; Kragh et al., 2024). The metagenomic assessment of drains highlighted that QAC tolerance genes are widely abundant in the FPEs with high numbers of genes associated with tolerance to QACs (*qac*G, *qac*J, and *qac*L, Figure 5B) We therefore propose that BC residuals left in the FPE may create niches, where increased tolerance to low concentrations might induce an important selective pressure, leading to better survival in subsequent biocide treatments. Such a scenario seemed to occur in the present study where the proportion of *Serratia* sp. increased in regrown biofilms and were among the dominating survivors identified after the second BC treatment in comparison to after the first treatment (Figure 7 and Figure 8). Such a clear example of selective pressure and improved survival due to previously applied biocide begs the question of whether rotation of biocides could prevent such one-sided development of the drain biofilm. In contrast, the usage of biocides such as SH and PAA with a broad mode of action left a more diverse biofilm with multiple genera surviving and regrowing. To the best of our knowledge, none of these biocides have been associated with specific biocide-tolerant genes.

## 5 Conclusion

In the present study, a biofilm drain model composed of 29 bacterial isolates was created based on culture-dependent and independent analysis of floor drains in different food processing environments. The biofilm model reflected >50% of the metagenomes of floor drains and >75% of the viable floor drain microbiome. While 274 different genera were identified, it was revealed that the drains were often dominated by few genera as only 22 genera were present at least one time at relative abundances >5%. *Pseudomonas* were the most frequently dominating genus with an average relative abundance of 18% and the most abundant genus in the viable microbiome. Biocide treatments of the biofilm model formed on SS coupons showed how PAA and SH were more effective in reducing the biofilm than BC. Repeated treatment with the same biocide did not lead to increased tolerance; however, biocides were selected for different bacterial genera in the surviving biofilm. This was most strikingly seen for the *S. marcescens* isolate, carrying a BC efflux pump (*oqxB*), as it became the most abundant bacteria in biofilms regrown following BC treatments. This showed how tolerance to low levels of residual biocides may provide a competitive advantage potentially leading to the domination of a niche within an FPE. Harborage of an efflux pump (*qacH*) in *L. monocytogenes* ST121 did not in contrast lead to an increase in abundance after BC treatments; however, this isolate survived in biofilms following all biocides treatments except the highest tested concentration of PAA, confirming the protective role biofilms have on the persistence of this pathogen in the FPE. Future studies should investigate whether rotational use of biocides can improve cleaning and disinfection regimes in the food industry as sequences of different biocides could be hypothesized to be able to control the biofilm better due to their different modes of action.

## Data Availability

The datasets generated for this study can be found in the European Nucleotide Archive (ENA) under project number: PRJEB72647.

## Footnotes

^1^ https://blast.ncbi.nlm.nih.gov/Blast.cgi

^2^ inkscape.org
