# Supplementary material for: Repeated biocide treatments cause changes to the microbiome of a food industry floor drain biofilm model
Source: Front Microbiol. 2025 Mar 14;16:1542193. doi: 10.3389/fmicb.2025.1542193 (PMC11949963; doi:10.3389/fmicb.2025.1542193)
Supplement: Supplementary file 1 [file Data_Sheet_1.zip › Supplementary Figure 1.docx]

Supplementary Material

Supplementary Figure S1


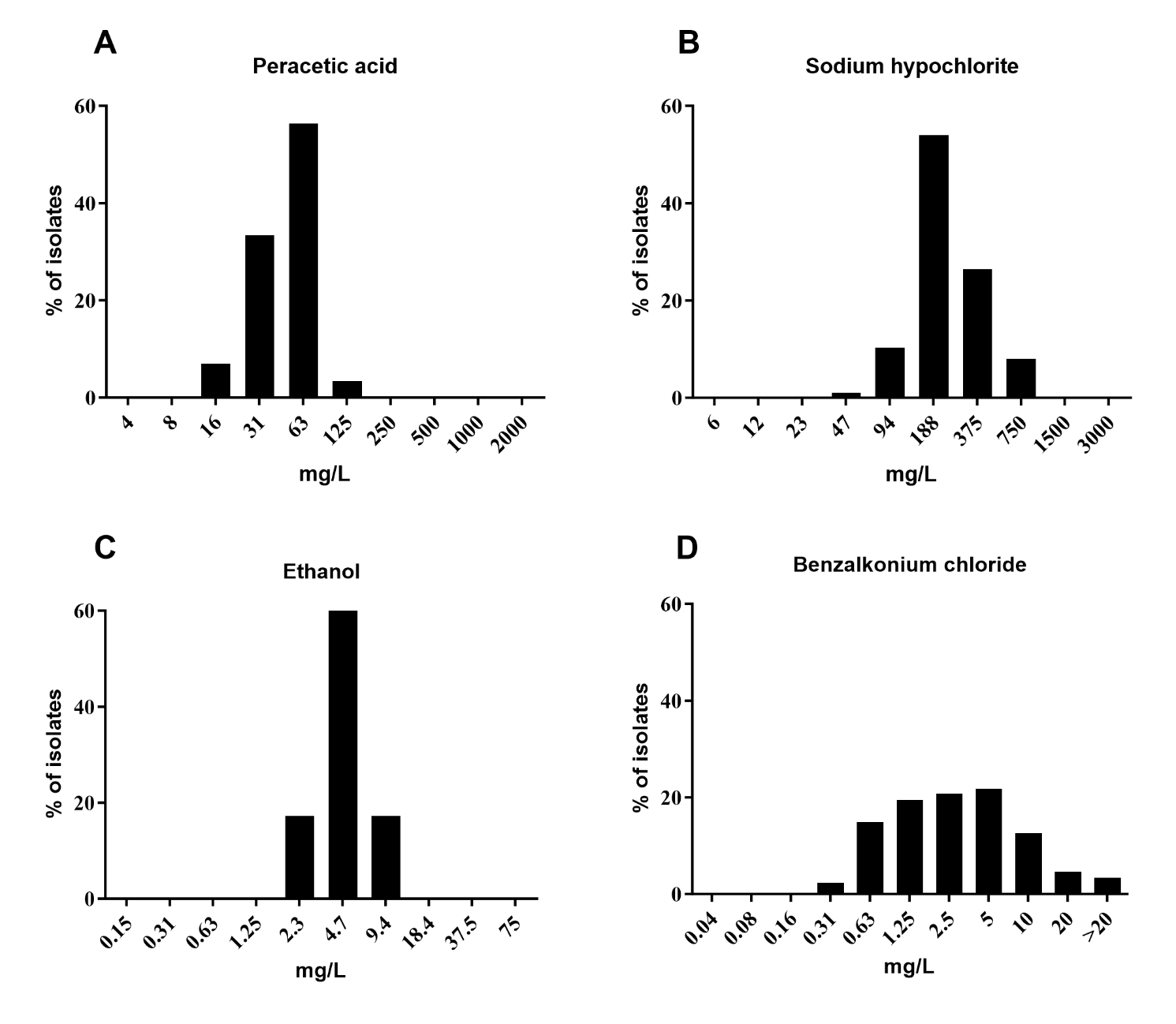


**Supplementary Figure S1.** Distribution of MIC-values for 87 representative drain isolates. MIC-values were determined in a minimum of two independent biological experiments with triplicates for the biocides A) peracetic acid, B) sodium hypochlorite, C), ethanol and D) benzalkonium chloride.
