# Supplementary material for: Repeated biocide treatments cause changes to the microbiome of a food industry floor drain biofilm model
Source: Front Microbiol. 2025 Mar 14;16:1542193. doi: 10.3389/fmicb.2025.1542193 (PMC11949963; doi:10.3389/fmicb.2025.1542193)
Supplement: Supplementary file 1 [file Data_Sheet_1.zip › Supplementary Figure 2.docx]

Supplementary Material

Supplementary Figure S2


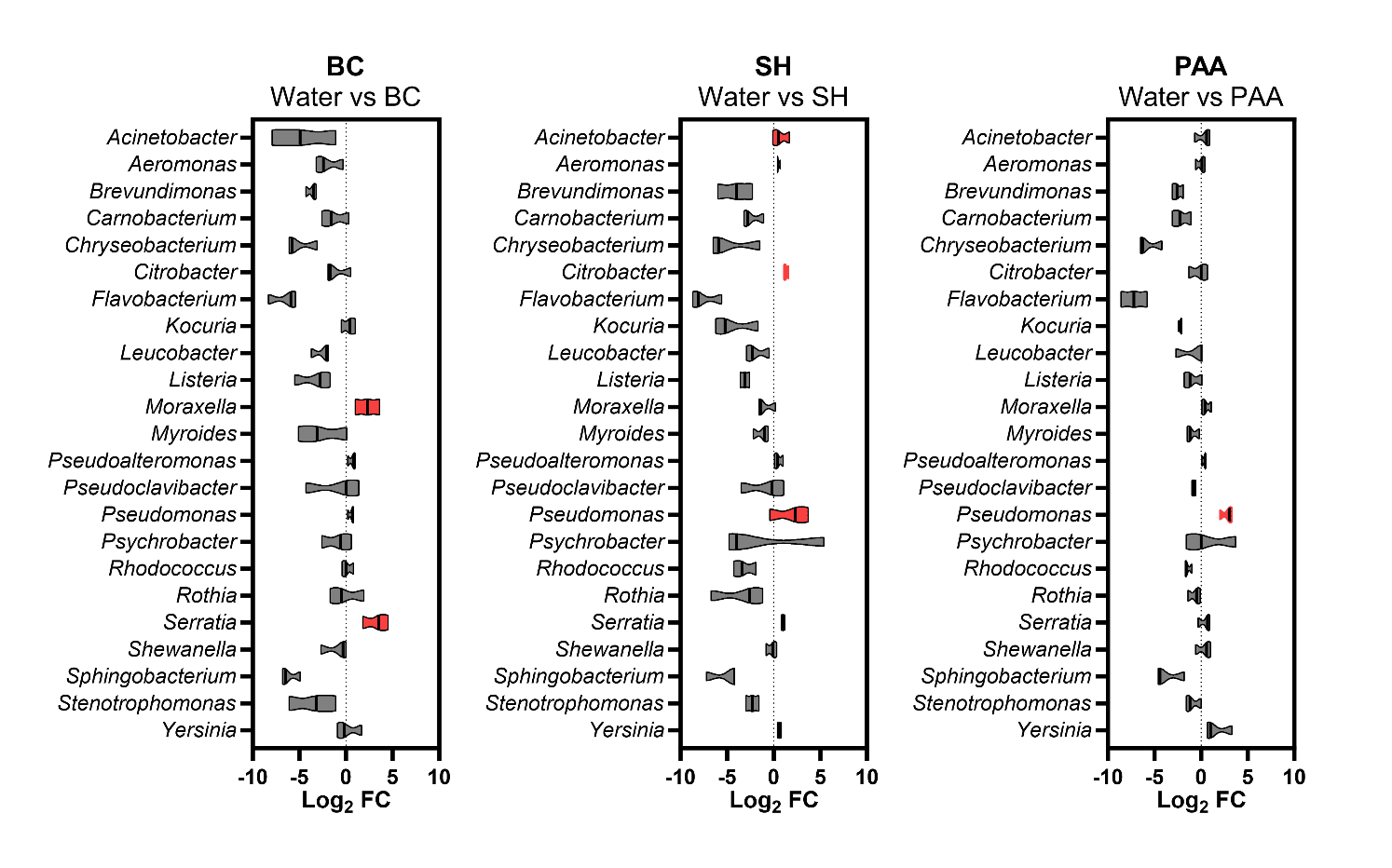


**Supplementary Figure S2.** Differential abundance analysis on changes in relative genus abundance of the regrown biofilm microbiome on day six after treatments with either benzalkonium chloride (BC), sodium hypochlorite (SH) or peracetic acid (PAA) on day three based on the comparison against the regrown control biofilm on day 6, which was treated with sterile water on day 3. The Log_2_ fold change depicted is the average of the fold change observed in each comparison of all three biocide concentrations (n=4), against the control (n=4). A) peracetic acid, B) sodium hypochlorite, C), ethanol and D) benzalkonium chloride. Genus boxes in red were significantly (P_adjusted_ < 0.05) increased in relative abundance compared to controls when all biocide concentrations (n=12), were compared against the control (n=4).
