# Supplementary material for: Repeated biocide treatments cause changes to the microbiome of a food industry floor drain biofilm model
Source: Front Microbiol. 2025 Mar 14;16:1542193. doi: 10.3389/fmicb.2025.1542193 (PMC11949963; doi:10.3389/fmicb.2025.1542193)
Supplement: Supplementary file 1 [file Data_Sheet_1.zip › Supplementary FIgure 3.docx]

Supplementary Material

Supplementary Figure S3


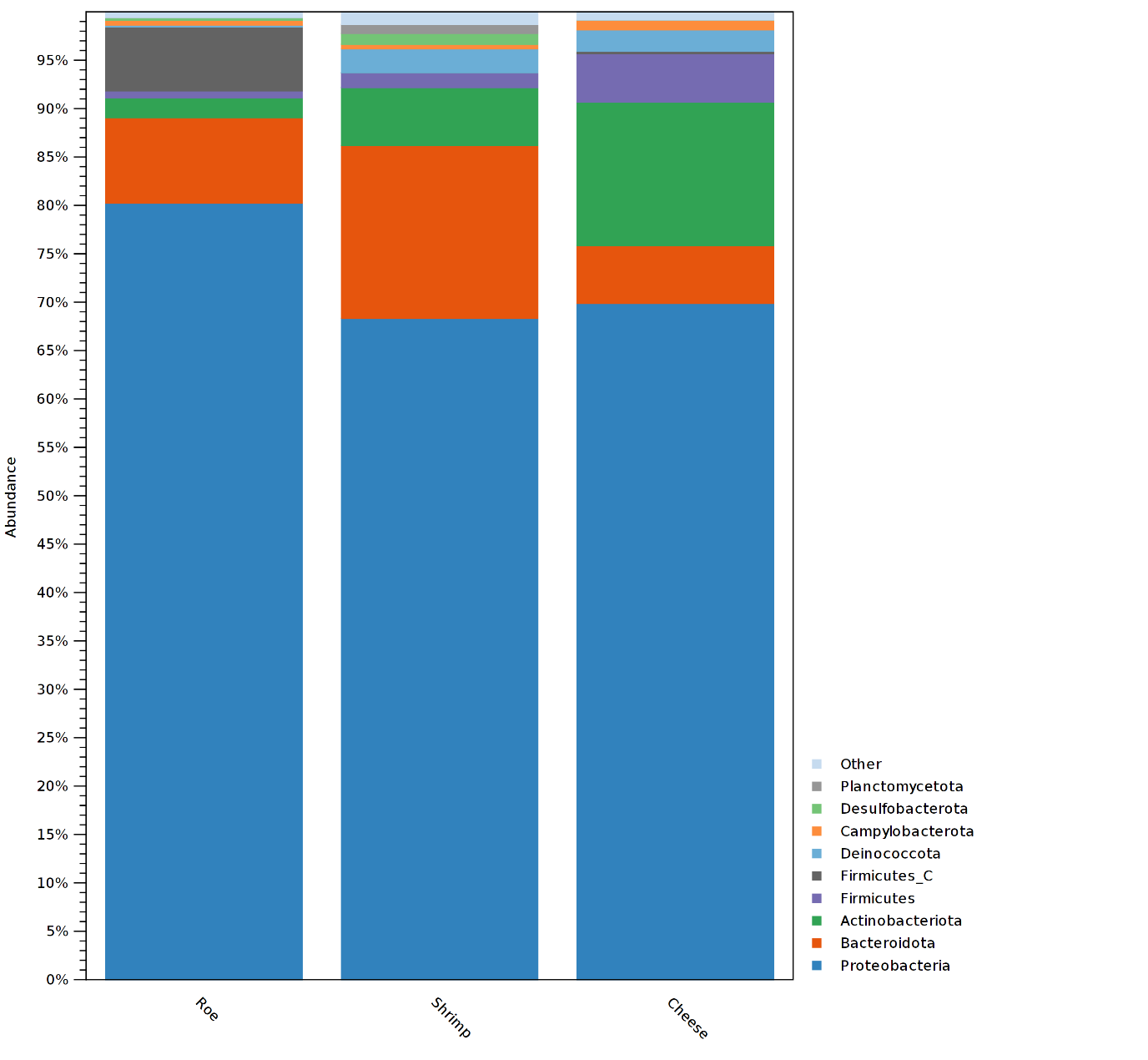


Supplementary Figure S3 Taxonomic profile of all 14 drains aggregated by food production environment. Cheese n=6, roe n=4, shrimp n=4. Aggregated by phylum. Since 2021 several phylum have been renamed: *Proteobacteria* is officially known as *Pseudomonadota,* *Actinobacteriota* as *Actinomycetota. Firmucutes* as *Bacillota.*
